# Automated classification of depression from structural brain measures across two independent community‐based cohorts

**DOI:** 10.1002/hbm.25095

**Published:** 2020-06-19

**Authors:** Aleks Stolicyn, Mathew A. Harris, Xueyi Shen, Miruna C. Barbu, Mark J. Adams, Emma L. Hawkins, Laura de Nooij, Hon Wah Yeung, Alison D. Murray, Stephen M. Lawrie, J. Douglas Steele, Andrew M. McIntosh, Heather C. Whalley

**Affiliations:** ^1^ Division of Psychiatry, University of Edinburgh Kennedy Tower, Royal Edinburgh Hospital, Morningside Park Edinburgh UK; ^2^ Aberdeen Biomedical Imaging Centre University of Aberdeen Lilian Sutton Building, Foresterhill Aberdeen UK; ^3^ School of Medicine (Division of Imaging Science and Technology) University of Dundee Dundee UK

**Keywords:** brain structure, classification, depression, diffusion MRI, machine learning, major depressive disorder, structural MRI

## Abstract

Major depressive disorder (MDD) has been the subject of many neuroimaging case–control classification studies. Although some studies report accuracies ≥80%, most have investigated relatively small samples of clinically‐ascertained, currently symptomatic cases, and did not attempt replication in larger samples. We here first aimed to replicate previously reported classification accuracies in a small, well‐phenotyped community‐based group of current MDD cases with clinical interview‐based diagnoses (from STratifying Resilience and Depression Longitudinally cohort, ‘STRADL’). We performed a set of exploratory predictive classification analyses with measures related to brain morphometry and white matter integrity. We applied three classifier types—SVM, penalised logistic regression or decision tree—either with or without optimisation, and with or without feature selection. We then determined whether similar accuracies could be replicated in a larger independent population‐based sample with self‐reported current depression (UK Biobank cohort). Additional analyses extended to lifetime MDD diagnoses—remitted MDD in STRADL, and lifetime‐experienced MDD in UK Biobank. The highest cross‐validation accuracy (75%) was achieved in the initial current MDD sample with a decision tree classifier and cortical surface area features. The most frequently selected decision tree split variables included surface areas of bilateral caudal anterior cingulate, left lingual gyrus, left superior frontal, right precentral and paracentral regions. High accuracy was not achieved in the larger samples with self‐reported current depression (53.73%), with remitted MDD (57.48%), or with lifetime‐experienced MDD (52.68–60.29%). Our results indicate that high predictive classification accuracies may not immediately translate to larger samples with broader criteria for depression, and may not be robust across different classification approaches.

## Graphical abstract

Previous case‐control classification studies of depression have reported promising accuracies up to 80% and above, but investigated relatively small samples with clinically‐ascertained cases. In the current study we explore multiple classification approaches with brain structural and white matter integrity measures, and achieve up to 75% predictive classification accuracy in a small sample with clinical interview‐based diagnoses (*N* < 50 cases, STRADL cohort). This accuracy, however, is not replicated in a larger sample of cases with self‐reported depression (*N* > 700 cases, UK Biobank cohort), and also in larger samples with remitted MDD (STRADL) and lifetime‐experienced MDD (UK Biobank). These results indicate that high predictive case‐control classification accuracies may not immediately translate to larger samples with broader diagnostic criteria for depression.

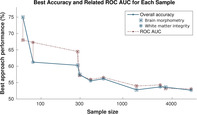

## 1 INTRODUCTION

### 1.1 Depression

Depression (major depressive disorder, MDD) is a prevalent psychiatric condition which on average affects between 10 and 15% of the general population over the lifetime in countries around the world (Bromet et al., 2011; Kessler & Bromet, 2013; Lim et al., 2018). It is among the leading causes of disability adjusted life years (Vos et al., 2012), and has been estimated to cost €92 billion in Europe and $210 billion in the USA in 2010 (Greenberg, Fournier, Sisitsky, Pike, & Kessler, 2015; Olesen et al., 2012). Depression remains a challenge to diagnose reliably and recent research indicates a particularly low rate of diagnostic agreement between specialists (Freedman et al., 2013). This emphasises the importance of developing better, more reliable and objective diagnostic methods for the illness.

### 1.2 Structural brain differences in depression

In depression, meta‐analyses report grey‐matter loss (compared to controls) in frontal and cingulate cortices, as well as subcortical structures including basal ganglia, thalamus, hippocampus and amygdala (Arnone et al., 2016; Arnone, McIntosh, Ebmeier, Munafò, & Anderson, 2012; Bora, Harrison, Davey, Yücel, & Pantelis, 2012; Kempton et al., 2011; Sacher et al., 2012; Wise et al., 2017). A recent multi‐site study with a large sample (*N* > 2,000 depression cases) also suggests significant thinning of cortical grey matter in orbitofrontal and cingulate cortices, as well as in the insula and temporal lobes in MDD patients (Schmaal et al., 2017).

With regard to white matter integrity, depression has been found to be associated with decreased fractional anisotropy (FA) in frontal, temporal and occipital brain regions, and more specifically in superior longitudinal fasciculus, uncinate fasciculus, anterior thalamus, medial forebrain bundle and corpus callosum (Bracht, Linden, & Keedwell, 2015; Chen et al., 2017; Jiang et al., 2017; Liao et al., 2013; Murphy & Frodl, 2011; Sexton, Mackay, & Ebmeier, 2009; van Velzen et al., 2019; Wen, Steffens, Chen, & Zainal, 2014). Lower FA and higher mean diffusivity (MD) are general indications of poorer white matter organisation and have been associated with depression, as well as of other psychiatric conditions (Shizukuishi, Abe, & Aoki, 2013).

### 1.3 Classification of depression with brain structural measures

One limitation of group‐level findings is that in most cases they cannot be applied directly for diagnostic purposes. This is because a single effect identified at group‐level may be present in some patients, but not in others. Moreover, brain regions are organised in networks (Sporns, 2013), and structural changes in one region can be related to changes in others (Calhoun, 2018; Xu, Groth, Pearlson, Schretlen, & Calhoun, 2009). It is hence more promising to combine multiple brain measures within a machine learning approach for a more accurate diagnostic detection (Arbabshirani, Plis, Sui, & Calhoun, 2017). This has been the subject of depression classification studies outlined below.

Existing studies have used measures derived from structural MRI and DTI, with depression classification accuracies ranging from 55% and up to and above 90% (reviews in Gao, Calhoun, & Sui, 2018; Kambeitz et al., 2017; Patel, Khalaf, & Aizenstein, 2016). Several studies with regional cortical thickness, surface area and volume measures reported cross‐validation accuracies between 75 and 80% (Kipli & Kouzani, 2015; Qiu et al., 2014). Although white matter integrity measures have seen limited application, several studies have also reported accuracies close to 75% (Matsuoka et al., 2017; Schnyer, Clasen, Gonzalez, & Beevers, 2017). Not all investigations have been as successful, however. In one study, for example, classification with subcortical grey matter volumes only reached 63% accuracy (Sacchet, Livermore, Iglesias, Glover, & Gotlib, 2015). In the most recent multi‐site study with independent training and test data sets, Yang et al. (2018) combined cortical and subcortical volume, cortical thickness and white matter integrity (FA) measures and achieved an accuracy of 75%. Specificity (percentage of correctly classified controls in the test sample), however, only reached modest 32% in this study—likely due to the smaller number of controls than cases. Samples in all studies were fairly small, with numbers of cases *N* < 60 (except for Yang et al., 2018, where data from *N* = 147 MDD cases were used for training and *N* = 83 for testing). In most studies case and control numbers were relatively balanced, with cases and controls also matched for age and sex. This was an exception for Yang et al. (2018), where there were three times more cases than controls.

An important limitation of the existing studies is that they did not investigate what accuracies can be achieved in larger samples with broader diagnostic criteria, but rather focused on relatively small samples with formally diagnosed depressed participants (Kambeitz et al., 2017). Depression is a very common condition and it is unclear whether high accuracies can generalise from clinically‐defined to community‐based samples, which are larger, more heterogeneous, and typically have less strict diagnostic criteria (Janssen, Mourão‐Miranda, & Schnack, 2018; Kim & Na, 2018; Schnack & Kahn, 2016).

### 1.4 Study aims

In the current study we first aimed to replicate previously reported accuracies in depression classification studies within a well‐characterised sample of formally diagnosed currently depressed participants with brain morphometric (cortical thickness, regional volumes and surface areas) and with white matter integrity (FA and MD) measures. We aimed to explore several classification techniques and brain measure subsets to identify the best accuracy that could be achieved in this well‐characterised data set, and to compare the accuracy with those of the previous studies. Samples in most previous studies were balanced, and hence we also aimed to analyse samples with balanced numbers of cases and controls, and with case and control participants matched for age and sex.

Our second aim was to determine if similar accuracies could be achieved in a larger (*N* > 700) independent sample with self‐reported current depression. Previous studies have only focused on small samples with formal diagnoses and in the current study we aimed to make a novel contribution by testing whether previous results can be replicated in a large community‐based cohort. Community‐based depression is the most prevalent and whether it can be accurately detected with brain measures remains an important open question, addressed in this study. In addition to investigating current depression, we aimed to explore what accuracies can be achieved in larger samples wit h either remitted or lifetime‐experienced depression. Previous literature did not focus on past depression and we aimed to fill this gap by testing whether classification accuracies in samples with these diagnostic criteria could be similar to those with criteria for current depression.

For each of the investigated diagnostic samples we aimed to test a number of alternative classification approaches in order to identify the best approach and the corresponding accuracy. Different classification methods are not equivalent and we considered that some may perform better than others on specific data sets (e.g., Fernández‐Delgado, Cernadas, Barro, & Amorim, 2014).

A more general aim of our study was to make a contribution to help bridge the gap between the depression research and the applied machine learning communities, as machine learning techniques are being increasingly used to investigate clinical populations (Arbabshirani et al., 2017; Janssen et al., 2018).

## 2 MATERIALS AND METHODS

### 2.1 Brain measure data sets

Brain measures were taken from two data sets: STRADL (Stratifying Depression and Resilience Longitudinally, Habota et al., 2019; Navrady et al., 2018) and UK Biobank (http://www.ukbiobank.ac.uk, RRID:SCR_012815; Bycroft et al., 2018; Sudlow et al., 2015). Brain morphometric measures (cortical thickness, cortical surface areas, cortical and subcortical volumes), alongside the relevant demographic information, were available for *N* = 622 participants from STRADL (quality controlled, scanned between June 2015 and August 2017), and for first and second releases of UK Biobank (*N* = 8,959 after quality control, January 2017 release). White matter integrity measures (FA and MD) were available for *N* = 873 participants from STRADL (quality controlled, scanned between June 2015 and January 2019) and for first through fourth releases of UK Biobank (*N* = 18,980 after quality control, October 2018 release). There were less participants with brain morphometric measures than with white matter integrity measures because derivation of brain morphometry data required more manual intervention during quality control in both cohorts. STRADL received ethical approval from the NHS Tayside committee on research ethics (reference 14/SS/0039). UK Biobank received ethical approval from the NHS Research Ethics Committee (reference 11/NW/0382), and the current study received approval from the UKB Access Committee (application #4844). All participants in both STRADL and UK Biobank gave written informed consent.

#### 2.1.1 Brain morphometric measures

In STRADL, T1‐weighted imaging was performed at two sites (Aberdeen and Dundee) with 3T magnetic resonance imaging scanners (Supplementary section S1.1). *N* = 650 acquired scans (*N* = 465 from Aberdeen and *N* = 185 from Dundee) were processed using FreeSurfer version 5.3 (http://surfer.nmr.mgh.harvard.edu, RRID:SCR_001847; Fischl, 2012) and quality‐controlled. *N* = 622 participants were included after quality control (please see Supplementary section S1.1.2 for details). Derived brain measures consisted of cortical thickness, cortical surface area and regional volumes for 34 bilateral cortical regions, as well as volumes for 21 subcortical regions (including four cerebellar regions) defined by the Desikan‐Killiany atlas (Desikan et al., 2006), comprising 225 measures in total.

In UK Biobank, T1‐weighted imaging data was collected at one site (Cheadle) with a 3T scanner (Siemens Skyra), following the standard and freely available UK Biobank imaging and quality control protocols (Alfaro‐Almagro et al., 2018; Smith, Alfaro‐Almagro, & Miller, 2018; UK Biobank, 2014). Brain morphometric measures for *N* = 10,109 T1‐weighted scans were derived locally with FreeSurfer version 5.3 and quality controlled (Supplementary section S1.1.3; Harris et al., 2019; Neilson et al., 2019; Ritchie et al., 2018). After quality control, measures for *N* = 8,959 participants were included.

#### 2.1.2 White matter integrity measures

In STRADL, diffusion‐weighted imaging was performed at the same two sites and with the same scanners as T1‐weighted imaging, as part of a single protocol. FA and MD measures were derived with FSL and TBSS toolkit for 896 participants (Tract‐Based Spatial Statistics, http://www.fmrib.ox.ac.uk/fsl, RRID:SCR_002823; Smith et al., 2006), following ENIGMA consortium protocols (http://enigma.ini.usc.edu/protocols/dti-protocols for FA measures and http://enigma.ini.usc.edu/protocols/dti-protocols/enigma-dti-diffusivity-protocol/ for MD measures). Average FA and MD measures were extracted for 19 bilateral and 5 unilateral tracts based on the Johns‐Hopkins University (JHU) white matter atlas (Mori & Crain, 2006)—this resulted in 43 FA and 43 MD measures in total for each participant. After quality control, *N* = 873 participants were included (Supplementary section S1.1.4).

In UK Biobank, diffusion‐weighted imaging was performed according to the standard UK Biobank imaging protocol (Smith et al., 2018, sections 2.8 and 2.10). FA and MD measures were derived for 21 bilateral tracts and six unilateral tracts, resulting in 48 FA and 48 MD measures in total (Mori & Crain, 2006; Smith et al., 2018, sections 3.10 and 3.10.1). UK Biobank protocol was slightly different from ENIGMA and there were measures for five more tracts compared to STRADL. Data for *N* = 18,980 participants were available after quality control by the UK Biobank and after outlier exclusion (Supplementary section S1.1.5).

#### 2.1.3 Correction for confounders

For STRADL data sets, covariates of no interest included age, sex and scan site. For UK Biobank data sets, covariates included age, sex, scan site (where appropriate) and three coordinates denoting head position in the scanner. Correction was performed by residualising each imaging measure separately over the potential confounder variables—fitting multiple linear regression model with covariates entered as predictors and the imaging measure as the response. Residuals of the fitted models were used as the corrected measures. In order to leverage the large cohort sizes, this correction was performed on the entire (quality‐controlled) data sets, prior to selection of case–control matched samples for classification.

### 2.2 Diagnostic criteria

A broad range of diagnostic criteria were assessed in order to evaluate different diagnostic frameworks and depths of phenotyping. Diagnoses of current or remitted depression in STRADL were based on clinical interviews and were derived following the DSM criteria (American Psychiatric Association, 2000). Diagnoses in UK Biobank were based either on self‐report (current or lifetime‐experienced symptoms), or on hospital records (lifetime‐experienced depression). For both STRADL and UK Biobank, we used two types of measures that respectively assessed (a) cross‐sectional, current depression symptoms, and (b) lifetime‐experienced or remitted depression. Whereas diagnostic criteria in STRADL were formal interview‐based, criteria in UK Biobank were more lenient as they were mainly based on self‐report (except for hospital‐recorded past depression, Table 1). Predictive modelling analyses were performed separately for each of the five diagnostic definitions across two brain measure domains (brain morphometry and white matter integrity), resulting in 10 sets of analyses in total. Further details of each of the diagnostic criteria are presented below and highlighted in Table 1.

**TABLE 1:** Summary of the main characteristics of the five investigated diagnostic criteria

	Diagnostic criteria	Current symptoms	Past symptoms	Assessment criteria	Assessment method	Cases (morphometry/white matter)
Current depression	cMDD‐STR	**✓**	‐	DSM	Clinical interview	30/40
	cMDD‐UKB	**✓**	**✓**	Manually defined	Self‐report	735/1,435
Past depression	rMDD‐STR	**✕**	**✓**	DSM	Clinical interview	148/202
	pMDD‐UKB CIDI	‐	**✓**	DSM	Self‐report	1,665/3,418
	pMDD‐UKB ICD	‐	**✓**	ICD	Clinical interview	140/289

*Note:* Tick symbol denotes symptoms were present, dash denotes symptoms could be either present or absent, cross symbol denotes symptoms were absent.

Abbreviations: cMDD‐STR, current MDD criteria in STRADL cohort; cMDD‐UKB, probable current MDD criteria in UK Biobank cohort; DSM, Diagnostic and Statistical Manual of Mental Disorders; ICD, International Statistical Classification of Diseases and Related Health Problems; pMDD‐UKB‐CIDI, lifetime MDD criteria based on the online Composite International Diagnostic Interview (CIDI) in UK Biobank cohort; pMDD‐UKB‐ICD, lifetime MDD criteria based on ICD and hospital records in UK Biobank cohort; rMDD‐STR, remitted MDD criteria in STRADL cohort.

#### 2.2.1 Diagnostic criteria in STRADL

Participants in STRADL were assessed with the research version of the Structured Clinical Interview for DSM Disorders (SCID, First, Gibbon, Spitzer, & Williams, 2002; Lobbestael, Leurgans, & Arntz, 2011). Diagnostic criteria for current MDD (cMDD‐STR) or remitted MDD (rMDD‐STR) were based on the diagnostic and statistical manual of mental disorders (DSM, American Psychiatric Association, 2000). Participants were considered remitted if they met criteria for at least one past episode of depression, but did not meet criteria for a current episode. Participants in STRADL cohort could meet criteria either for current or remitted MDD, but not for both.

#### 2.2.2 Diagnostic criteria in UK biobank

In UK Biobank, no formal clinical assessment of depression was made at the time of the scan. We hence defined participants who were likely symptomatic at scan‐time based on the criteria defined in Smith et al. (2013), combined with self‐reported current symptoms. Briefly, participants were classed as having probable current MDD (cMDD‐UKB) if they reported low mood or lack of interest lasting 2 weeks at any time in the past (single‐episode or recurrent), history of seeing a psychiatrist or a GP for nerves, anxiety, tension or depression, and reported current symptoms relevant to depression according to a screening assessment at the time of the scan (see Supplementary section S1.2.2 for screen and exclusion details; Smith et al., 2013; UK Biobank, 2011). Participants were excluded if they had any major co‐morbid neurological or psychiatric disorder—schizophrenia, bipolar, multiple personality disorder, autism, intellectual disability, Parkinson's disease, multiple sclerosis or cognitive impairment.

There were two diagnostic definitions related to lifetime experience of MDD. The first definition was based on the questions from Composite International Diagnostic Interview (CIDI‐SF, Kessler, Andrews, Mroczek, Ustun, & Wittchen, 1998), which was administered as part of the UK Biobank online mental health questionnaire at a subsequent time after the imaging assessment (Davis et al., 2019; UK Biobank, 2017). Briefly, participants were classed as having had lifetime experience of MDD (past MDD, pMDD‐UKB‐CIDI) if they reported experiencing one or more depressive episodes in their life according to the DSM criteria (American Psychiatric Association, 2000). Participants were excluded from control sample for pMDD‐UKB‐CIDI definition if they were likely to have experienced depression (Supplementary section S1.2.3). Assessment in pMDD‐UKB‐CIDI definition was similar to SCID, but administered as part of an online questionnaire.

The second diagnostic definition for lifetime MDD was derived from medical records and was based on a formal past diagnosis of depression, made by a clinician in a hospital setting according to the ICD criteria (pMDD‐UKB‐ICD; UK Biobank, 2019; World Health Organisation, 1992; Supplementary section S1.2.4). Because medical records were not available for all participants in the UK Biobank, some pMDD‐UKB‐ICD cases may have been missed. Participants were excluded from control sample for this definition if they self‐reported past experience of mood disorder.

Two definitions of ‘lifetime‐experienced’ MDD were studied because we assumed that there could be differences between self‐reported and formally clinically‐assessed experience of depression. The main difference between the diagnostic criteria for lifetime experience of MDD (UK Biobank) and remitted MDD (STRADL) was that participants meeting lifetime criteria could have an ongoing episode, while those with remitted MDD could not. Our rationale was to check if slight differences in assessment and inclusion criteria between these three samples with past depression could lead to different classification outcomes. It should be noted that participants in UK Biobank could meet criteria for more than one diagnostic definition—cases in pMDD‐UKB‐CIDI and pMDD‐UKB‐ICD, as well as cMDD‐UKB samples could overlap between each other.

### 2.3 Matched sample selection

Selection of age and sex matched cases and controls was performed primarily to enable balanced class data for classifier training and testing. Tables 2 and 3 outline the numbers and main demographic characteristics of participants who met criteria for each of the five defined diagnostic definitions, with brain morphometric and white matter integrity measures respectively (between *N* = 140 and *N* = 3,418 cases in each sample). For each case participant from cMDD‐STR and rMDD‐STR samples, we selected a control with no history of depression, matched by handedness, sex and scan site, and with the smallest difference in age. It is worth highlighting that control samples were drawn to have minimal difference with cases with regard to demographic criteria (particularly age) and were thus non‐random. For each case participant from UK Biobank (cMDD‐UKB, pMDD‐UKB‐CIDI and pMDD‐UKB‐ICD samples), we selected a control with the same sex and scan site, and the smallest age difference. Importantly, there were *N* > 700 cases with self‐reported current depression in UK Biobank (cMDD‐UKB sample), which is significantly more than in the previous depression classification studies (Gao et al., 2018; Kambeitz et al., 2017).

**TABLE 2:** Summary demographic information for cases and controls in the five analysed samples with *brain morphometric measures* (cortical thickness, surface areas, and volumes)

	Sample	Characteristic	Controls	Cases
Current depression	cMDD‐STR	Size	30	30
		Sex (male/female)	8/22	8/22
		Age (years)	54.23 (10.98)	54.07 (10.96)
		QIDS	3.3 (2.25)	13.97 (3.59)
		Medicated	2	18
	cMDD‐UKB	Size	735	735
		Sex (male/female)	215/520	215/520
		Age (years)	59.66 (7.21)	59.66 (7.21)
Past depression	rMDD‐STR	Size	148	148
		Sex (male/female)	44/104	44/104
		Age (years)	58.06 (8.02)	57.16 (8.81)
		QIDS	3.49 (2.35)	5.48 (3.91)
	pMDD‐UKB‐CIDI	Size	1,665	1,665
		Sex (male/female)	544/1,121	544/1,121
		Age (years)	60.91 (7.18)	60.90 (7.19)
	pMDD‐UKB‐ICD	Size	140	140
		Sex (male/female)	49/91	49/91
		Age (years)	61.59 (7.64)	61.59 (7.65)

*Note:* Standard deviations for age and QIDS are in brackets. In cMDD‐STR sample participants were considered medicated if they had at least one antidepressant prescription. Abbreviations: cMDD‐STR, current MDD criteria in STRADL cohort; cMDD‐UKB, probable current MDD criteria in UK Biobank cohort; pMDD‐UKB‐CIDI, lifetime MDD criteria based on the online Composite International Diagnostic Interview (CIDI) in UK Biobank cohort; pMDD‐UKB‐ICD, lifetime MDD criteria based on ICD and hospital records in UK Biobank cohort; QIDS, Quick Inventory of Depressive Symptomatology; rMDD‐STR, remitted MDD criteria in STRADL cohort.

**TABLE 3:** Summary demographic information for cases and controls in the five analysed samples with *white matter integrity measures* (FA and MD)

	Sample	Characteristic	Controls	Cases
Current depression	cMDD‐STR	Size	40	40
		Sex (male/female)	10/30	10/30
		Age (years)	55.03 (9.77)	54.23 (10.35)
		QIDS	3.58 (2.54)	14.13 (3.88)
		Medicated	2	28
	cMDD‐UKB	Size	1,435	1,435
		Sex (male/female)	451/984	451/984
		Age (years)	60.11 (7.16)	60.11 (7.16)
Past depression	rMDD‐STR	Size	202	202
		Sex (male/female)	56/146	56/146
		Age (years)	57.89 (8.84)	56.97 (9.28)
		QIDS	3.53 (2.36)	5.38 (3.68)
	pMDD‐UKB‐CIDI	Size	3,418	3,418
		Sex (male/female)	1,094/2,324	1,094/2,324
		Age (years)	61.46 (7.09)	61.44 (7.11)
	pMDD‐UKB‐ICD	Size	289	289
		Sex (male/female)	97/192	97/192
		Age (years)	61.77 (7.70)	61.76 (7.70)

*Note:* Standard deviations for age and QIDS are in brackets. In cMDD‐STR sample participants were considered medicated if they had at least one antidepressant prescription. Abbreviations: cMDD‐STR, current MDD criteria in STRADL cohort; cMDD‐UKB, probable current MDD criteria in UK Biobank cohort; pMDD‐UKB‐CIDI, lifetime MDD criteria based on the online Composite International Diagnostic Interview (CIDI) in UK Biobank cohort; pMDD‐UKB‐ICD, lifetime MDD criteria based on ICD and hospital records in UK Biobank cohort; QIDS, Quick Inventory of Depressive Symptomatology; rMDD‐STR, remitted MDD criteria in STRADL cohort.

### 2.4 Predictive modelling

Predictive modelling was performed separately with brain morphometric data and with white matter integrity data—there were therefore 10 matched case–control data sets (five diagnostic definitions across two feature domains). For each of the 10 data sets we performed either leave‐one‐out (LOOCV), 10‐fold or fivefold cross‐validation, depending on the size of the data set. Cross‐validation was attempted separately with three classifier models, with different feature subdomains (e.g., all brain morphometric measures or only cortical thickness, surface area, volume or subcortical measures), with or without classifier hyperparameter optimisation, and with or without feature selection (e.g., Patel et al., 2015; Qiu et al., 2014; Schnyer et al., 2017; Yang et al., 2018). Where feasible, cross‐validation was repeated multiple times with different fold partitions. Cross‐validation accuracies, sensitivities, specificities and area under receiver operating characteristic curve (ROC AUC, Melo, 2013) were recorded for each analysis to identify classification approaches with the best results.

#### 2.4.1 Classification models and optimisation

We explored three classification models—support vector machine with a Gaussian kernel (SVM, Cortes & Vapnik, 1995; Hofmann, Schölkopf, & Smola, 2008), penalised logistic regression (PLR, Zou & Hastie, 2005), and the simple decision tree (DT, Kingsford & Salzberg, 2008). SVM was chosen because of wide use of the technique in previous neuroimaging classification studies with some success (Arbabshirani et al., 2017; Kambeitz et al., 2017; Patel et al., 2016). PLR was selected because it is a linear classifier and has been shown to perform well in some previous studies with neuroimaging data (e.g., Dadi et al., 2019). DT was applied because of its low computational complexity and suitability for data sets with relatively small numbers of features. Classifier training and testing was performed with MATLAB R2015b Statistics and Machine Learning Toolbox (http://www.mathworks.com/products/matlab/, Mathworks Inc, RRID:SCR_001622).

Classification with SVM and DT classifiers was attempted both with and without hyperparameter optimisation. PLR model always requires hyperparameter optimisation. Further details on the specified fixed hyperparameter values and hyperparameter search grids can be found in Supplementary sections S1.3.2 and S1.3.3.

#### 2.4.2 Feature selection

Two feature selection methods were attempted with SVM and decision tree classifiers. The methods were a *t*‐test filter and a wrapper method based on sequential feature elimination (Aha & Bankert, 1996; Mwangi, Tian, & Soares, 2014). Filter feature selection is widely used in neuroimaging classification studies (Kambeitz et al., 2017; Mwangi et al., 2014; Patel et al., 2016), while sequential feature elimination was applied because it offers more extensive exploration of feature space compared to other methods, and counts of features in our study (less than 250 in all analyses) enabled its application with reasonable computation times. Sequential feature elimination is very computationally expensive and we only applied it with fixed sets of hyperparameters (no combined hyperparameter optimisation). In PLR classification, feature selection is already embedded through elastic net regularisation (Zou & Hastie, 2005) and no additional feature selection was performed.

Sequential feature elimination was not performed in cMDD‐UKB and pMDD‐UKB‐CIDI samples with combined brain morphometric feature set and decision tree classifier, due to large sample sizes and high computational complexity (Supplementary section S2.2). Further details on the applied feature selection methods can be found in Supplementary sections S1.3.4 and S1.3.5.

#### 2.4.3 Cross‐validation

In analyses with cMDD‐STR diagnostic definition we applied LOOCV due to the small data set size (*N* = 60 participants with morphometric brain measures and *N* = 80 participants with white matter integrity measures)—in order to maximise the amount of training data. In all other analyses we used 10‐fold cross‐validation, with an exception for pMDD‐UKB‐CIDI data set of white matter integrity measures. pMDD‐UKB‐CIDI white matter integrity data set was the largest (*N* = 6,836 participants) and we applied fivefold cross‐validation to enable classifier training and optimisation in reasonable time. Cross‐validation was repeated 10 times with pre‐determined random fold partitions for each classification approach in smaller data sets (rMDD‐STR and pMDD‐UKB‐ICD diagnostic criteria). This was not feasible for the larger data sets due to long optimisation times (cMDD‐UKB and pMDD‐UKB‐CIDI diagnostic criteria), and hence cross‐validation was performed only once with a single predefined partition. Fold partitions for the larger data sets were deterministically defined with an algorithm which aimed to maximally balance cross‐validation folds with respect to age and sex (Supplementary section S1.3.6).

#### 2.4.4 Comparison of classification methods

To compare classification approaches we applied either corrected paired *t*‐tests (rMDD‐STR and pMDD‐UKB‐ICD data sets, Bouckaert & Frank, 2004; Nadeau & Bengio, 2003), or McNemar's test (cMDD‐UKB and pMDD‐UKB‐CIDI data sets, McNemar, 1947). Each approach was given a relative score according to the number of approaches which performed worse. Further details on comparison of classification methods can be found in Supplementary section S1.3.7.

#### 2.4.5 Case–control differences

In addition to predictive modelling, we checked for case–control differences in the 10 evaluated samples using simple two‐sample *t* tests with corrections for false discovery rate (Benjamini & Hochberg, 1995). Results for these analyses are reported in Supplementary section S2.1.

## 3 RESULTS

To summarise, accuracies above 60% were only achieved in the small current MDD sample from STRADL (best accuracy 75% with brain morphometric features and 61.25% with white matter integrity features, Tables 4 and 5). Best accuracies across all classification attempts in samples with all other diagnostic criteria were between 52.68 and 60.29%, and are summarised in Table 6.

**TABLE 4:** Case‐control classification accuracies and ROC AUC measures (on cross‐validation) with brain morphometric features in cMDD‐STR sample (30 cases and 30 controls)

Classifier type	Feature selection	Hyperparam. optimisation	Outer CV	Inner CV	Feature domain	Classification accuracy (sensitivity/specificity)	ROC AUC
PLR	Embedded	Grid search	LOOCV	10‐fold	Thickness	*60.00% (56.67/63.33%)*	0.609
					Surface area	51.67% (50.00/53.33%)	0.547
					Volume	56.67% (60.00/53.33%)	0.539
					Subcortical	55.00% (56.67/53.33%)	0.572
					Combined	50.00% (46.67/53.33%)	0.546
SVM	None	None	LOOCV	‐	Thickness	*63.33% (63.33/63.33%)*	0.682
					Surface area	46.67% (43.33/50.00%)	0.529
					Volume	51.67% (40.00/63.33%)	0.550
					Subcortical	60.00% (60.00/60.00%)	0.582
					Combined	61.67% (70.00/53.33%)	0.568
		Grid search		LOOCV	Thickness	60.00% (60.00/60.00%)	0.556
					Surface area	50.00% (46.67/53.33%)	0.500
					Volume	61.67% (50.00/73.33%)	0.649
					Subcortical	58.33% (50.00/66.67%)	0.602
					Combined	58.33% (63.33/53.33%)	0.628
	Statistical filter	None			Combined	53.33% (40.00/66.67%)	0.540
		Grid search		10‐fold	Combined	45.00% (40.00/50.00%)	0.513
	Sequential elimination	None			Thickness	61.67% (56.67/66.67%)	0.687
					Surface area	48.33% (43.33/53.33%)	0.519
					Volume	50.00% (36.67/63.33%)	0.556
					Subcortical	53.33% (50.00/56.67%)	0.573
					Combined	61.67% (63.33/60.00%)	0.659
DT	None	None	LOOCV	‐	Thickness	38.33% (40.00/36.67%)	0.283
					Surface area	*75.00% (80.00/70.00%)*	0.680
					Volume	45.00% (43.33/46.67%)	0.377
					Subcortical	43.33% (46.67/40.00%)	0.394
					Combined	55.00% (50.00/60.00%)	0.473
		Grid search		LOOCV	Thickness	51.67% (53.33/50.00%)	0.318
					Surface area	65.00% (76.67/53.33%)	0.677
					Volume	46.67% (46.67/46.67%)	0.442
					Subcortical	58.33% (56.67/60.00%)	0.500
					Combined	38.33% (46.67/30.00%)	0.407
	Statistical filter	None			Combined	33.33% (43.33/23.33%)	0.189
		Grid search		10‐fold	Combined	43.33% (43.33/43.33%)	0.350
	Sequential elimination	None			Thickness	35.00% (46.67/23.33%)	0.255
					Surface area	68.33% (70.00/66.67%)	0.637
					Volume	40.00% (40.00/40.00%)	0.292
					Subcortical	51.67% (46.67/56.67%)	0.426
					Combined	63.33% (56.67/70.00%)	0.533

*Note:* Top accuracies for SVM, PLR and DT classifiers are in italics.

Abbreviations: CV, cross‐validation; DT, decision tree; LOOCV, leave‐one‐out cross‐validation; PLR, penalised logistic regression; ROC AUC, receiver operating characteristic area under the curve; SVM, support vector machine.

**TABLE 5:** Case‐control classification accuracies and ROC AUC measures (on cross‐validation) with white matter integrity features in the cMDD‐STR sample (40 cases and 40 controls)

Classifier type	Feature selection	Hyperparam optimisation	Outer CV	Inner CV	Feature domain	Classification accuracy (sensitivity/specificity)	ROC AUC
PLR	Embedded	Grid search	LOOCV	10‐fold	FA	31.25% (35.00/27.50%)	0.363
					MD	53.75% (55.00/52.50%)	0.589
					Combined	48.75% (50.00/47.50%)	0.474
SVM	None	None	LOOCV	‐	FA	48.75% (40.00/57.50%)	0.484
					MD	57.50% (55.00/60.00%)	0.536
					Combined	52.50% (50.00/55.00%)	0.520
		Grid search		LOOCV	FA	50.00% (40.00/60.00%)	0.505
					MD	*61.25% (57.50/65.00%)*	0.673
					Combined	53.75% (52.50/55.00%)	0.559
	Statistical filter	None			FA	40.00% (32.50/47.50%)	0.345
					MD	37.50% (30.00/45.00%)	0.353
					Combined	30.00% (20.00/40.00%)	0.283
		Grid search		10‐fold	FA	52.50% (60.00/45.00%)	0.476
					MD	38.75% (40.00/37.50%)	0.385
					Combined	38.75% (40.00/37.50%)	0.328
	Sequential elimination	None			FA	47.50% (40.00/55.00%)	0.488
					MD	53.75% (52.50/55.00%)	0.534
					Combined	51.25% (55.00/47.50%)	0.501
DT	None	None	LOOCV	‐	FA	53.75% (50.00/57.50%)	0.434
					MD	56.25% (55.00/57.50%)	0.514
					Combined	*57.50% (42.50/72.50%)*	0.552
		Grid search		LOOCV	FA	48.75% (65.00/32.50%)	0.350
					MD	47.50% (40.00/55.00%)	0.372
					Combined	51.25% (47.50/55.00%)	0.563
	Statistical filter	None			FA	45.00% (35.00/55.00%)	0.323
					MD	43.75% (30.00/57.50%)	0.331
					Combined	36.25% (30.00/42.50%)	0.256
		Grid search		10‐fold	FA	42.50% (47.50/37.50%)	0.280
					MD	40.00% (35.00/45.00%)	0.204
					Combined	33.75% (35.00/32.50%)	0.267
	Sequential elimination	None			FA	48.75% (45.00/52.50%)	0.433
					MD	52.50% (52.50/52.50%)	0.458
					Combined	56.25% (55.00/57.50%)	0.488

*Note:* Top accuracies for SVM, PLR and DT classifiers are in italics.

Abbreviations: CV, cross‐validation; DT, decision tree; LOOCV, leave‐one‐out cross‐validation; PLR, penalised logistic regression; ROC AUC, receiver operating characteristic area under the curve; SVM, support vector machine.

**TABLE 6:** *Best* accuracies and related ROC AUC measures for case‐control classification (on cross‐validation) for brain moprhometric and white matter integrity features in cMDD‐UKB, rMDD‐STR, pMDD‐UKB‐CIDI, and pMDD‐UKB‐ICD samples

Data set	Feature domain	Sample size	Classification approach	Classification accuracy (sensitivity/specificity)	ROC AUC
cMDD‐UKB	Cortical thickness features	1,470	PLR classifier ‐ Hyperparameter grid search ‐ Embedded feature selection	52.80% (52.66/52.92%)	0.540
	Combined FA and MD features	2,870	SVM classifier ‐ No hyperparameter optim. ‐ Sequential feat. Elimination	53.73% (51.08/56.37%)	0.549
rMDD‐STR	Combined brain morphometric features	296	Decision tree classifier ‐ Hyperparameter grid search ‐ Filter feature selection	*57.48% (52.57/62.35%)*	0.572
	MD features	404	SVM classifier ‐ Hyperparameter grid search ‐ No feature selection	55.54% (59.16/51.92%)	0.560
pMDD‐UKB‐CIDI	Cortical thickness features	3,330	SVM classifier ‐ No hyperparameter optim. ‐ No feature selection	53.63% (53.72/53.54%)	0.532
	Combined FA and MD features	6,836	SVM classifier ‐ *5‐fold inner/outer CV* ‐ No hyperparameter optim. ‐ Sequential feat. Elimination	52.68% (53.63/51.73%)	0.531
pMDD‐UKB‐ICD	Combined brain morphometric features	280	PLR classifier ‐ Hyperparameter grid search ‐ Embedded feature selection	*60.29% (61.86/58.71%)*	0.645
	MD features	578	SVM classifier ‐ No hyperparameter optim. ‐ Filter feature selection	56.18% (68.56/43.83%)	0.566

*Note:* Combined brain morphometric features included cortical thickness, surface area, cortical and subcortical volume measures. Nested 10‐fold outer and 10‐fold inner cross‐validation was performed in all analyses, except where otherwise specified. Top two accuracies are in italics.

Abbreviations: cMDD‐UKB, sample with probable current MDD in UK Biobank cohort; FA, fractional anisotropy; MD, mean diffusivity; PLR, penalised logistic regression; pMDD‐UKB‐CIDI, sample with lifetime MDD based on the online Composite International Diagnostic Interview (CIDI) criteria in UK Biobank cohort; pMDD‐UKB‐ICD, sample with lifetime MDD based on the ICD criteria and hospital records in UK Biobank cohort; rMDD‐STR, sample with remitted MDD in STRADL cohort; ROC AUC, receiver operating characteristic area under the curve; SVM, support vector machine.

### 3.1 Classification of current MDD and controls

**FIGURE 1:**
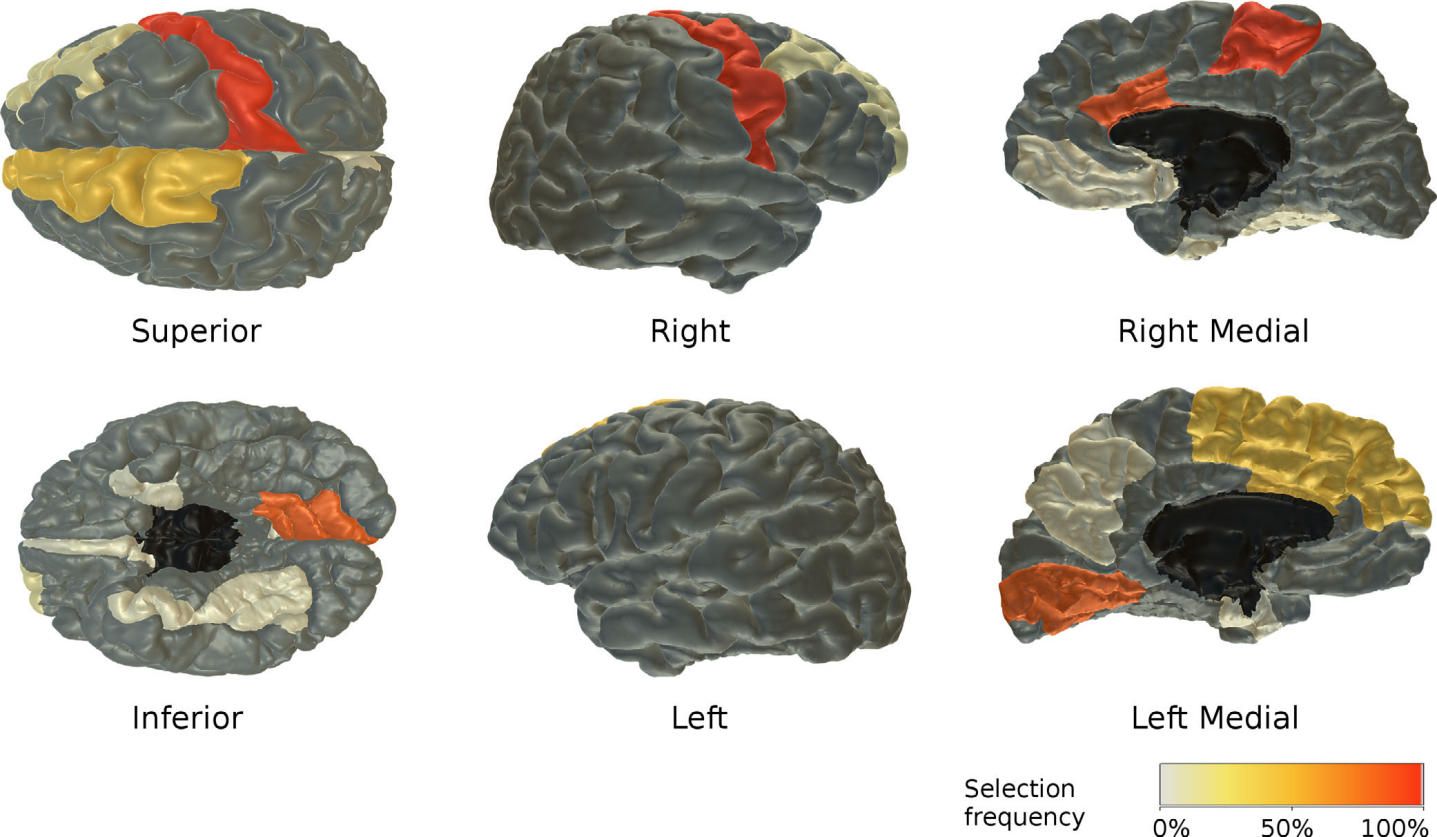
Surface area regions consistently selected as decision tree cut features across cross‐validation folds in cMDD‐STR sample. Colour of each region indicates fraction of folds where surface area of the region was selected as one of the cut variables. Regions in dark grey were never selected. Most frequently selected regions include bilateral caudal anterior cingulate, left lingual gyrus, left superior frontal, right precentral and paracentral regions

Top classification accuracy in the small cMDD‐STR sample was 75% (sensitivity 80%, specificity 70%, ROC AUC 0.68) with all surface area features and the simple decision tree classifier, no hyperparameter optimisation, and no feature selection. Importance of each feature in this analysis can be defined by the fraction of cross‐validation folds where the feature was selected as one of decision tree cut variables. Surface area features with highest contribution according to this criteria are illustrated in Figure 1—these included right paracentral and precentral regions (selected in all folds), right caudal anterior cingulate (54 of 60 folds), left lingual gyrus (51 of 60 folds), left caudal anterior cingulate (28 of 60 folds), and left superior frontal region (26 of 60 folds). We additionally assessed feature contributions to the best classification accuracy by excluding one feature at a time and assessing drops in cross‐validation accuracy. Top contributing features according to this criteria were the four most important in Figure 1—right precentral (accuracy drop 23.3%), right paracentral (20%), left lingual (10%) and right caudal anterior cingulate (8.3%) regions.

Classification accuracy decreased from 75 to 68.33% (sensitivity 70.00%, specificity 66.67%, ROC AUC 0.637) with added sequential feature elimination and with hyperparameter optimisation it reached 65% (sensitivity 76.67%, specificity 53.33%, ROC AUC 0.677). This could be indicative of optimisation‐related over‐fitting. Accuracies with all other feature domains with DT classifier were below 60%. With SVM classifier, top accuracy reached 63.33% with all thickness features (no optimisation or feature selection; sensitivity 63.33%, specificity 63.33%, ROC AUC 0.682). Accuracies for all classification attempts with morphometric brain measures in cMDD‐STR sample can be found in Table 4.

For white matter integrity measures, top accuracy reached 61.25% (sensitivity 57.50%, specificity 65%, ROC AUC 0.673) with SVM classifier, all MD features and hyperparameter grid search. Accuracies for all other optimisation attempts were below 58% and are outlined in Table 5.

We performed additional analyses to check if the best classification accuracy can be replicated with a different set of controls. The alternative set of controls was again matched to cases by age and sex, although matching for age was slightly worse compared to the original sample. The previous best approach (decision tree with surface area features) with added sequential feature elimination reached 61.67% accuracy (sensitivity 63.33%, specificity 60%, ROC AUC 0.501), which was the best for the sample. Similar accuracy was achieved with SVM with hyperparameter grid search and cortical thickness features (sensitivity 60%, specificity 63.33%, ROC AUC 0.57), and with PLR classifier and combined feature set (sensitivity 63.33%, specificity 60%, ROC AUC 0.648). Results for the original best approach without added sequential feature elimination were not replicated (accuracy 50%, sensitivity 60%, specificity 40%, ROC AUC 0.438). Details of these analyses can be found in Supplementary section S2.2.

### 3.2 Classification of self‐reported current depression and controls in large UKB sample

High classification accuracies were not replicated in the large population‐based sample with self‐reported current depression (cMDD‐UKB). Top accuracy for the data set with brain morphometric measures only reached 52.80% (sensitivity 52.66%, specificity 52.92%, ROC AUC 0.540) with PLR classifier (hyperparameter optimisation and embedded feature selection) and cortical thickness features. Top accuracy with white matter integrity measures was 53.73% (sensitivity 51.08%, specificity 56.37%, ROC AUC 0.549) with SVM classifier, combined FA and MD features and sequential feature elimination. Accuracies and scores for each classification approach can be found in Tables S12 and S16.

### 3.3 Classification of remitted MDD and controls

Top accuracy in rMDD‐STR sample with brain morphometric measures reached 57.48% (sensitivity 52.57%, specificity 62.35%, ROC AUC 0.572) with decision tree classifier, hyperparameter grid search, filter feature selection and combined feature set (best approach score of 13 of maximal 37). This was closely followed by decision tree without optimisation or feature selection (accuracy 57.09%, sensitivity 56.64%, specificity 57.47%, ROC AUC 0.591, score 11). Accuracies for all other classification attempts with brain morphometric measures were lower than 56% and can be found alongside the related scores in Table S11.

Top accuracy with white matter integrity measures was 55.54% (sensitivity 59.16%, specificity 51.92%, ROC AUC 0.560) with SVM, all MD features and hyperparameter grid search (classification approach score second‐best with 6 of 28). This was closely followed by PLR classifier with accuracy 55.15% (sensitivity 53.48%, specificity 56.78%, ROC AUC 0.560, score 7), and SVM with no optimisation or feature selection (accuracy 55.08%, sensitivity 54.23%, specificity 55.90%, score 6). Accuracies for all other classification attempts were below 55% and can be found in Table S15.

### 3.4 Classification of lifetime‐experienced MDD and controls

#### 3.4.1 Classification of self‐reported lifetime MDD and controls

Top accuracy in pMDD‐UKB‐CIDI sample with brain morphometric features was 53.63% (sensitivity 53.72%, specificity 53.54%, ROC AUC 0.532) with SVM classifier, no optimisation or feature selection and cortical thickness features. Top accuracy with white matter integrity measures was only 52.68% (sensitivity 53.63%, specificity 51.73%, ROC AUC 0.531) with SVM and sequential feature elimination on combined set of FA and MD measures. Accuracies for all other classification attempts were lower and can be found alongside scores for each approach in Tables S13 and S17.

#### 3.4.2 Classification of hospital‐recorded lifetime MDD and controls

Top accuracy for the pMDD‐UKB‐ICD sample with brain morphometric measures reached 60.29% (sensitivity 61.86%, specificity 58.71%, ROC AUC 0.645) with PLR classifier and combined feature set (best score of 20 out of maximal 37). Accuracies for all other classification attempts with brain morphometric measures were below 59% and can be found alongside scores for each approach in Table S14.

Top accuracy with white matter integrity measures was 56.18% (sensitivity 68.56%, specificity 43.83%, ROC AUC 0.566) with SVM classifier, MD features and filter feature selection (best score of 12 of maximal 31). All other accuracies for the sample were below 56% and can be found together with scores for each approach in Table S18.

## 4 DISCUSSION

### 4.1 Classification of current depression

#### 4.1.1 Classification accuracy

The best classification accuracies were achieved in the small sample with formally‐diagnosed current MDD (cMDD‐STR, 75% with decision tree and surface area features; 61.25% with MD features and SVM classifier with optimised hyperparameters). These results are broadly consistent with those of previous studies with similar feature domains, where best accuracies were between 60 and 80% (Kipli & Kouzani, 2015; Matsuoka et al., 2017; Qiu et al., 2014; Sacchet et al., 2015; Schnyer et al., 2017; Yang et al., 2018). MD features in the study appeared more discriminative of current MDD than FA features (Table 5), which is consistent with some of the previous work in our lab (Barbu et al., 2019; Shen et al., 2019).

It could be highlighted that combination of feature subsets almost never outperforms the subsets applied individually, even with feature selection or regularisation (Tables 4 and 5). This could be because the feature subsets contain more non‐overlapping redundant information compared to complementary depression‐relevant information (for example volume measures depend on both surface area and cortical thickness measures), and thus combining them does not aid in classification.

The best accuracy of 75% was not replicated when the control sample was replaced, although a similar classification approach achieved an accuracy of 61.67% (Table S19). This could in part be due to the fact that the replaced controls were slightly less well matched to cases with respect to age. On the other hand, it is likely that non‐depressed control participants are heterogeneous and some may be better discriminated from depressed participants than others.

#### 4.1.2 Predictive brain regions

Six surface area measures were identified as the most predictive of current MDD (Figure 1). Decreases in grey matter in precentral cortex were previously reported in several studies (Grieve, Korgaonkar, Koslow, Gordon, & Williams, 2013; Zhang et al., 2012), although reductions in surface area in this region were reported more specifically for adolescent depression (Schmaal et al., 2017). Anterior cingulate cortex has long been theorised to play an important role in MDD due to its involvement in processing of reward and emotional information (Diener et al., 2012; Holroyd & Umemoto, 2016; Rolls, 2016); brain structural studies do indeed show changes in this region, as well as in the adjacent superior frontal cortex (Grieve et al., 2013; Li et al., 2020; Schmaal et al., 2017; Zhao et al., 2017). For lingual gyrus, increased grey‐matter volume was reported in late‐life and late‐onset depression (Ancelin et al., 2019; Du et al., 2014), but surface area reductions were found in adolescent depression (Schmaal et al., 2017). These regions are, of course, only a subset of those identified as altered in MDD and others include the wider frontal cortex and the subcortical structures including the amygdala, hippocampus and the thalamus (Arnone et al., 2016; Wise et al., 2017). It is likely that the current MDD cases in our study represent a subtype of depression characterised by changes in the identified regions and that other subtypes may be characterised by different patterns of changes in the brain.

### 4.2 Classification of self‐reported current depression in larger population sample

The core novel contribution of our study is in attempted classification of depression in the comparatively very large community‐based UK Biobank sample (cMDD‐UKB). There were *N* = 735 cases in the data set of brain morphometric measures and *N* = 1,435 cases in the data set of white matter integrity measures (Tables 2 and 3), which is significantly larger than in most previous depression classification studies (Gao et al., 2018; Kambeitz et al., 2017). High accuracies were not replicated in this sample (Tables 6, S12 and S16) which indicates that community‐based depression, which is the most prevalent, cannot be accurately detected using structural brain measures.

**FIGURE 2:**
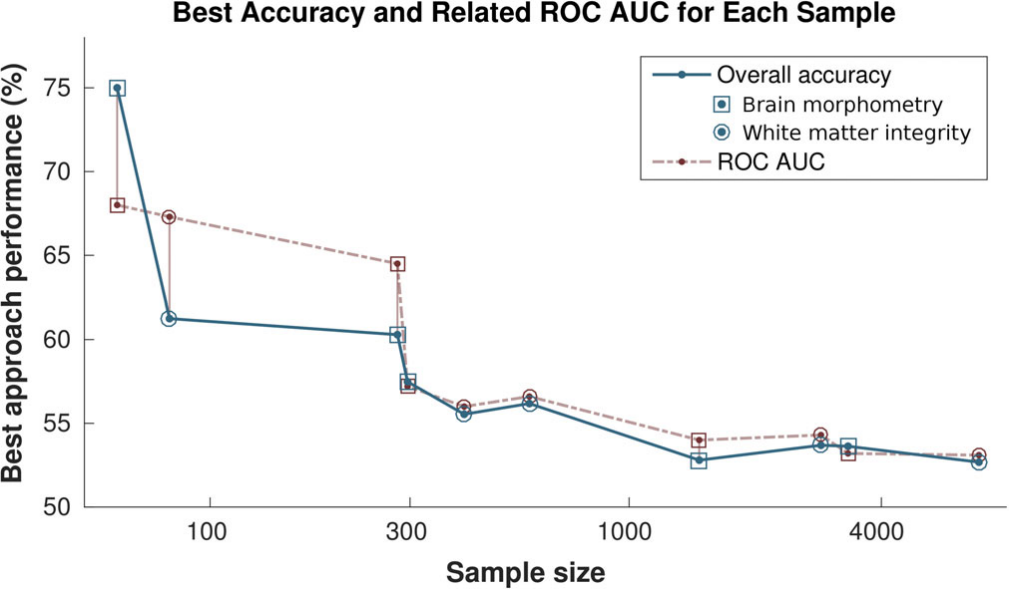
Best classification accuracies plotted against sample sizes for all 10 analysed samples (five diagnostic definitions across two feature domains—brain morphometry and white matter integrity). Best accuracy tended to decrease towards chance level with increasing sample size. Sample size/abscissa axis logarithmically scaled. Abbreviation: ROC AUC, receiver operating characteristic area under the curve

Consistent with our findings, recent reviews indicate that the highest accuracies to date have only been achieved in imaging classification studies with small samples (*N* < 100 participants), and that accuracy tends to decrease with larger sample sizes (Arbabshirani et al., 2017; Janssen et al., 2018). Kim and Na (2018) highlight that because most studies focus on small and relatively homogeneous samples, best results may not immediately translate to real‐world settings with large heterogeneous depressed populations, due to factors such as co‐morbidities, medication, differences in illness severity and recurrence, and clinical subtypes. In addition, other factors such as multiple scanning sites and difficulties with managing artefacts in large data sets may also be at play (e.g., Johnston, Mwangi, Matthews, Coghill, & Steele, 2013). Our results underscore importance of these points—high accuracies were only found in the small cMDD‐STR sample, were not replicated in larger samples, and best accuracies tended to decrease towards chance level with increasing sample size (Figure 2).

Apart from the large sample size and the resulting heterogeneity, one factor which may have contributed to the lower accuracies is the difference in the diagnostic criteria. There were no formal diagnoses at scan time for UK Biobank participants and the current depression diagnoses were based on self‐reported past and current symptoms (Supplementary section S1.2.2). These criteria arguably correspond to less severe forms of depression, more prevalent in community settings. Less severe depression is likely to have fewer and weaker associations with changes in brain structure, which in turn may have contributed to lower classification accuracies.

### 4.3 Classification of remitted or lifetime‐experienced depression

The second novel contribution of our study is attempted classification of depression in samples with diagnostic criteria related to past depression (remitted or lifetime‐experienced). Top two accuracies were 60.29% for pMDD‐UKB‐ICD diagnostic definition, and 57.48% for rMDD‐STR definition, with all brain morphometric features (Table 6). Previous classification literature did not study past depression, but indicates that the best accuracies can be achieved in samples with severe and chronic/treatment‐resistant ongoing episodes (e.g., Johnston, Steele, Tolomeo, Christmas, & Matthews, 2015; Mwangi, Ebmeier, Matthews, & Steele, 2012). These are typically patients who are medicated and seen long‐term in clinical care, but who are studied less often due to long time and resources necessary for recruitment. In a recent review, Kambeitz et al. (2017) highlight that current symptom severity (as measured by Hamilton Depression Rating Scale, Hamilton, 1980) could predict better classification outcomes across 33 analysed studies, covering both brain structural and functional feature modalities (e.g., task‐related and resting‐state brain activation; Johnston et al., 2015; Zeng et al., 2012). Our results extend the previous literature and suggest that, compared to current depression, cases with past depression are even more difficult to discriminate from healthy controls based on structural brain measures.

It is worth highlighting that participants with past depression in our study were of relatively older age (mean ages 57–62, Tables 2 and 3). With increasing age brain structure may be influenced more by medications, cardio‐vascular health, lifestyle and other factors, which may in turn impact how depression affects the brain. Future research could take these factors into account when classifying past or present depression in older age.

### 4.4 Classification methods

Selection of classification methods in our study was guided by the previous neuroimaging literature and computational complexity considerations. SVM, decision tree and penalised logistic regression are among the most promising classifiers based on the previous studies (Arbabshirani et al., 2017; Dadi et al., 2019; Fernández‐Delgado et al., 2014; Kambeitz et al., 2017). Sequential feature elimination enabled the most extensive feasible exploration of the feature space, but is computationally expensive and could only be applied in our study because of the relatively low numbers of features (225 or less in all analyses). Future studies could attempt classification with other data modalities such as VBM or multiple estimates of connectivity between brain regions. Because feature counts in these modalities are typically very large, alternative classification methods could include neural network classifiers, minimum redundancy maximum relevance (mRMR) feature selection, or recursive feature elimination (Ramírez‐Gallego et al., 2017; Sanz, Valim, Vegas, Oller, & Reverter, 2018; Vieira, Pinaya, & Mechelli, 2017).

## 5 CONCLUSION

We explored a range of classification approaches with brain morphometric and white matter integrity measures, and were able to achieve cross‐validation accuracies up to 75% in the small sample with formally‐defined current MDD. The core contribution of our study is that these results could not be replicated in a comparatively very large community‐based sample with self‐reported depression. High classification accuracies were also not replicated in larger samples with remitted or lifetime‐experienced MDD. Previous studies largely focused on small samples with formal diagnoses for current depression. Our results complement this literature and suggest that it may not be possible to accurately detect community‐based depression in large samples with structural brain measures. Future studies could examine whether high accuracies can be achieved in larger samples with formally diagnosed and more severe MDD (for example, long‐term psychiatric outpatients), and explore other feature domains such as task‐related fMRI and brain connectivity.

## CONFLICT OF INTERESTS

Over the past 3 years, S. M. L. received research grant support from Janssen, as well as personal fees from Sunovion and Janssen. J. D. S previously received research funding from Wyeth and Indivior. A. M. M. previously received research grant support from The Sackler Trust, as well as speaker fees from Illumina and Janssen. H. C. W. previously received research grant support from Pfizer. None of these funding sources are connected to the present study. No potential conflicts of interest are reported for other authors.

## Supporting information


**Appendix** **S1:** Supporting information

## Data Availability

The data collected in the STRADL study have been incorporated in the larger Generation Scotland dataset. Non‐identifiable information from the Generation Scotland cohort is available to researchers in the United Kingdom and to international collaborators through application to the Generation Scotland Access Committee (access@generationscotland.org) and through the Edinburgh Data Vault (https://doi.org/10.7488/8f68f1ae‐0329‐4b73‐b189‐c7288ea844d7). Generation Scotland operates a managed data access process including an online application form, and proposals are reviewed by the Generation Scotland Access Committee. Data from the UK Biobank resource is available for health‐related research upon registration and application through the UK Biobank Access Management System (https://www.ukbiobank.ac.uk/register‐apply/).
