# Supplementary material for: Automated classification of depression from structural brain measures across two independent community‐based cohorts
Source: Hum Brain Mapp. 2020 Jun 19;41(14):3922–37. doi: 10.1002/hbm.25095 (PMC7469862; doi:10.1002/hbm.25095)
Supplement: Supplementary file 1 — Appendix S1: Supporting information [file HBM-41-3922-s001.pdf]

Automated Classification of Depression from Structural Brain Measures across Two Independent Community-based Cohorts

Supplementary Material

Aleks Stolicyn[1], Mathew A. Harris[1], Xueyi Shen[1], Miruna C. Barbu[1], Mark J. Adams[1], Emma L. Hawkins[1], Laura de Nooij[1], Hon Wah Yeung[1], Alison D. Murray[2], Stephen M. Lawrie[1], J. Douglas Steele[3], Andrew M. McIntosh[1], Heather C. Whalley[1]

1. Division of Psychiatry, University of Edinburgh
   Kennedy Tower, Royal Edinburgh Hospital, Morningside Park
   Edinburgh EH10 5HF, United Kingdom.

2. Aberdeen Biomedical Imaging Centre, University of Aberdeen
   Lilian Sutton Building, Foresterhill, Aberdeen AB25 2ZD, United Kingdom.

3. School of Medicine (Division of Imaging Science and Technology)
   University of Dundee, Dundee DD1 9SY, United Kingdom.

Corresponding author:

Aleks Stolicyn
Division of Psychiatry, Centre for Clinical Brain Sciences, University of Edinburgh
Kennedy Tower, Royal Edinburgh Hospital, Morningside Park, Edinburgh EH10 5HF, UK.
Email: a.stolicyn@ed.ac.uk

S1. MATERIALS AND METHODS

S1.1 Brain Imaging

S1.1.1 Scanning Details

Brain imaging and scanning sequence details for UK Biobank participants were previously described elsewhere (please see Alfaro-Almagro et al., 2018; Smith, Alfaro-Almagro, & Miller, 2018; UK Biobank, 2014). We here describe the T1-weighted and DTI scanning sequence details for STRADL participants, scanned in Aberdeen and in Dundee.

Aberdeen participants in STRADL were imaged on a 3T Philips Achieva TX-series MRI system (Philips Healthcare, Best, Netherlands) with a 32 channel phased-array head coil with a back facing mirror (software version 5.1.7; gradients with maximum amplitude 80 mT/m and maximum slew rate 100 T/m/s). For T1-weighted imaging, 160 sagittal slices were acquired with repetition time 8.3 ms, echo time 3.8 ms, inversion time 1031 ms, 8˚ flip angle, field of view 240 mm, matrix size 240 × 240, and voxel size 0.9 × 0.9 × 1.0 mm^3. Total acquisition time was 5 minutes and 38 seconds. For DTI imaging, there were 60 axial slices with repetition time 7010 ms, echo time 90 ms, 90˚ flip angle, field of view 220 mm, matrix size 96 × 94, voxel size 2.3 × 2.3 × 2.3 mm^3, 64 non-collinear gradient directions (b = 1200 s/mm^2), and eight diffusion unweighted images. Total acquisition time was 9 minutes and 28 seconds.

Dundee participants in STRADL were imaged using a Siemens 3T Prisma-FIT scanner (Siemens Healthineers, Erlangen, Germany) with 20 channel head and neck coil and a back facing mirror (software version VE11, gradient with maximum amplitude 80 mT/m and maximum slew rate 200 T/m/s). 208 sagittal slices were acquired with repetition time of 1740 ms, echo time 2.62 ms, inversion time of 900 ms, 8˚ flip angle, field of view 256 mm, matrix

size 256 × 256, and voxel size 1.0 × 1.0 × 1.0 mm^3 . Acquisition time was 4 minutes and 3 seconds. For DTI imaging, 60 axial slices were acquired with repetition time 7100 ms, echo time 87 ms, 90˚ flip angle, field of view 220 mm, matrix size 96 × 94, voxel size 2.3 × 2.3 × 2.3 mm^3, 64 non-collinear gradient directions (b = 1200 s/mm2), and eight diffusion unweighted images. Acquisition time was 8 minutes and 54 seconds.

S1.1.2 STRADL FreeSurfer Processing Details

T1-weighted scans for N = 650 participants were processed with FreeSurfer version 5.3. The FreeSurfer processed scans were visually inspected and minor errors were manually corrected. Errors included incorrect skull stripping, exclusion of grey or white matter in tissue segmentation maps, or incorrect brain parcellation into separate regions (Neilson et al., 2019, supplementary material). Participants were excluded when there was at least one major error that could not be corrected or when there were multiple minor errors (N = 6). Additional N = 16 participants had at least one cortical measure missing after processing and were also excluded. As an additional quality control step, we also excluded N = 6 participants who were more than three standard deviations different from the sample mean in at least one of three global cortical measures – range (standard deviation) of cortical thickness across brain regions, sum of cortical region volumes, or sum of regional surface areas. N = 622 individuals were available for the STRADL dataset of brain morphometric measures (Figure S1A).

S1.1.3 UK Biobank FreeSurfer Processing Details

T1-weighted scans for N = 10,109 participants were processed with FreeSurfer version 5.3. Participants were excluded in cases of general FreeSurfer processing failure, one or more major processing errors, or multiple minor errors as described above for STRADL (N = 1,029). We additionally excluded N = 121 participants as outliers in global cortical metrics

(as above for STRADL), resulting in a dataset of N = 8,959 subjects in total (Figure S2A).

S1.1.4 STRADL DTI Processing Details

Diffusion-weighted images for 980 participants were corrected for eddy current-related distortions and head movements (‘*eddy_correct*’ function in FSL), which was followed by skull stripping and computation of FA and MD maps. Skull stripping was performed with BET with a threshold of 0.2. FA and MD images were computed with DTIFIT component of FSL (https://fsl.fmrib.ox.ac.uk/fsl/fslwiki/FDT/UserGuide#DTIFIT). As part of the ENIGMA protocol, images were first slightly eroded to remove brain-edge artefacts, and then nonlinearly registered to the ENIGMA template and transformed into 1 × 1 × 1 mm standard space. N = 12 participants with FA image distortions or poor template registration were excluded after visual inspection. White matter skeleton was calculated as the mean of all registered FA images. FA data for each participant was then projected onto the skeleton with a threshold of FA > -0.049. Tracts for FA and MD measure ROI extraction were based on the Johns-Hopkins University (JHU) DTI-based white matter atlas (Mori & Crain, 2006). At the time of the study demographic data was available for N = 884 of N = 968 processed participants. As an additional quality control step, we excluded participants where global FA or global MD measures were more than three standard deviations different from the entire sample means. We here consider first principal components for all 43 FA and MD measures as representative of global FA and MD. Outlier exclusion resulted in data for N = 873 participants being available for STRADL dataset (Figure S1B).

S1.1.5 UK Biobank DTI Processing Details

As part of the UK Biobank DTI processing protocol, diffusion-weighted images were corrected for head motion and eddy currents and processed with the TBSS toolkit to extract FA and MD skeletons (Smith et al., 2006; Smith et al., 2018, sections 3.10 and 3.10.1). FA

and MD measures were derived from skeletons for 21 bilateral tracts and 6 unilateral tracts. Data for N = 19,393 participants were available. Similar to the STRADL data, outliers in global FA and MD measures were excluded, which resulted in N = 18,980 participants remaining in the final dataset (supplementary Figure S2B).

S1.2 Diagnostic Criteria

S1.2.1 STRADL Diagnostic Criteria

As described in the main text, diagnoses for STRADL participants were established using Structured Clinical Interview for DSM Disorders (SCID), and were based on the Diagnostic and Statistical Manual of Mental Disorders (American Psychiatric Association, 2000; First, Gibbon, Spitzer, & Williams, 2002). Participants were classed as currently depressed (cMDD-STR) if they had an ongoing MDD episode, and as remitted (rMDD-STR) if they had at least one past episode of MDD, but were not depressed at the time of the scan.

S1.2.2 UK Biobank Probable Current MDD (cMDD-UKB) Diagnostic Definition

Criteria for probable current MDD (cMDD-UKB) in UK Biobank was based on the three diagnostic categories defined in Smith et al. (2013), combined with a screen of symptoms at the time of the scan. Briefly, the categories in Smith et al. (2013) were based on self-reported past symptoms of depression (low mood or anhedonia lasting for at least two weeks at any time in their life), and self-reported history of seeing a psychiatrist or a GP for nerves, anxiety, tension or depression. Based on the self-reported participant data, Smith et al. defined three diagnostic categories – single-episode, moderate recurrent or severe recurrent past (lifetime) depression. We classed participants as cMDD-UKB if they met criteria for either of the three categories, and also reported current symptoms. Participants screened positive for current symptoms if they fulfilled at least one of the following criteria:

1) Reported depressed mood over the past two weeks for more than half of the days or

nearly every day (UKB touchscreen questionnaire, data item #2050);

2) Reported lack of interest or pleasure in daily activities over the past two weeks for more than half of the days or nearly every day (UKB touchscreen questionnaire, data item #2060);

3) Reported in general feeling very unhappy or extremely unhappy (UKB touchscreen questionnaire, data item #4526);

4) Reported at least one symptom for at least three of four symptom groups related to depression – mood symptoms, sleep problems, psychomotor symptoms or interpersonal symptoms.

Mood symptoms mentioned above included the following items:

– Often feeling miserable (UKB touchscreen questionnaire, data item #1930);

– Often feeling fed-up (UKB touchscreen questionnaire, data item #1960);

– Experiencing depressed mood for several days over the past two weeks (UKB touchscreen questionnaire, data item #2050);

– Experiencing lack of interest or pleasure for several days over the past two weeks (UKB touchscreen questionnaire, data item #2060);

– Feeling moderately unhappy in general (UKB touchscreen questionnaire, data item #4526).

Sleep problems were defined by the following items:

– Experiencing difficulty getting up in the morning (UKB touchscreen questionnaire, data item #1170);

– Usually experiencing trouble in falling asleep, or waking up in the middle of the night (UKB touchscreen questionnaire, data item #1200).

Psychomotor symptoms included the following items:

– Often experiencing restlessness over the past two weeks (UKB touchscreen questionnaire, data item #2070);

– Experiencing tiredness or lack of energy nearly every day over the past two

weeks (UKB touchscreen questionnaire, data item #2080).

Interpersonal symptoms were defined by the following items:

– Often being irritable (UKB touchscreen questionnaire, data item #1940);
– Experiencing hurt feelings easily (UKB touchscreen questionnaire, data item #1950);
– Often feeling lonely (UKB touchscreen questionnaire, data item #2020);
– Often being troubled by feelings of guilt (UKB touchscreen questionnaire, data item #2030).

Participants were excluded from both cases and controls if they reported having Parkinson's disease, bipolar disorder, multiple personality disorder, schizophrenia, autism, intellectual disability, multiple sclerosis or cognitive impairment. Participants were also excluded from control samples if they reported depression, anxiety or other mood disorder, use of anxiolytic, antidepressant or antipsychotic drugs, had nervous breakdown or suicide attempt in the past, or had seen a GP or a psychiatrist about nerves, anxiety or depression.

S1.2.3 UK Biobank CIDI-SF Lifetime MDD (pMDD-UKB-CIDI) Diagnostic Definition

Composite International Diagnostic Interview assessment (CIDI-SF, Kessler, Andrews, Mroczek, Ustun, & Wittchen, 1998) was administered in UK Biobank as part of an online mental health questionnaire (UK Biobank, 2017). Participants were first asked if they had ever experienced a period of two weeks in which they had low or depressed mood, or a lack of interest or pleasure in daily activities. If they responded positively to either of the two questions, they were asked six additional questions about whether they experienced other symptoms of depression according to the DSM criteria at the same time (American Psychiatric Association, 2000). The assessed symptoms were related to feelings of worthlessness, tiredness, difficulty in concentrating, thoughts of death, changes in weight, and changes in sleeping patterns (UK Biobank, 2017). Participants were classed as having

had lifetime experience of MDD if they met all of the following criteria:

– Experienced at least five of the eight depression symptoms at the same time
– Experienced low mood or lack of interest every day or almost every day during the episode, with feelings lasting most of the day or all day
– Reported a level of psychosocial impairment (study / employment / childcare / housework or leisure) during the episode

Participants were excluded from being controls for pMDD-UKB-CIDI definition if they reported a diagnosis of depression or had a score above 5 in PHQ-9 according to the online assessment (Kroenke & Spitzer, 2002).

S1.2.4 UK Biobank ICD Lifetime MDD (pMDD-UKB-ICD) Diagnostic Definition

Some participants in UK Biobank had a formal past diagnosis of depression, established by a clinician, reported in their hospital record. The diagnosis was established according to the ICD (World Health Organisation, 1992), but was only available for participants who were depressed during a hospital admission. These participants were classed as pMDD-UKB-ICD cases. Participants who did not have a hospital record available were not included as either cases or controls. Participant who were included as controls may have been depressed at some point in their life, but not during a hospital admission.

S1.3 Classification Methods

S1.3.1 Classification Model Details

SVM with Gaussian kernel, decision tree and penalised logistic regression classifiers were selected because they performed relatively well in previous neuroimaging classification studies (Arbabshirani, Plis, Sui, & Calhoun, 2017; Dadi et al., 2019; Kambeitz et al., 2017; Yang et al., 2018), and because these classifiers have been reported among the most promising across different datasets (Fernández-Delgado, Cernadas, Barro, & Amorim, 2014).

Penalised logistic regression was applied with *elastic net* penalty, which has performed relatively well in a previous study (Yang et al., 2018; Zou & Hastie, 2005). Splitting criterion for the decision tree classifier was Gini's Diversity Index, which is the default criterion in MATLAB R2015b. Features used for SVM and PLR classifier training and testing were standardised – centred by feature means and scaled by standard deviations in the training data. Classification was attempted both with and without hyperparameter optimisation for SVM and DT classifiers, and only with optimised hyperparameters for PLR classifier.

S1.3.2 Fixed Hyperparameters

SVM classifier has two main hyperparameters – *box constraint* (regularisation) and *kernel scale*. When no optimisation was applied, box contraint was set to the canonical value of 1, which the default heuristic implemented in MATLAB R2015b and other machine learning toolkits (Chang & Lin, 2011; Pedregosa et al., 2018). Kernel scale parameter was set to the square root of the number of features for each dataset, which is the heuristic implemented in LibSVM toolkit (Chang & Lin, 2011). Rationale for this heuristic is that the optimal kernel scale depends on the distance between data points from different classes, which is in turn bounded by the number of features, when the features are standardised.

Decision tree classifier has three core hyperparameters – *maximum number of splits, minimum parent size*, and *minimum leaf size.* Maximum number of splits was set to 20 and minimum parent size was set to 10 following the default MATLAB R2015b heuristics for medium-sized trees. There was no MATLAB heuristic for the minimum leaf size and this parameter was set to the value of 4, following the heuristic implemented in 'rpart' R package (Therneau, Atkinson, & Ripley, 2019). The 'rpart' package heuristic suggests specifying the minimum leaf size as $\frac{1}{3}$ of the minimum parent size to reduce possibilities for overfitting.

PLR classifier always requires optimisation of the *regularisation coefficient* (lambda) and hence analyses with fixed hyperparameters were not attempted.

S1.3.3 Hyperparameter Optimisation

Hyperparameters were optimised through grid search with inner cross-validation accuracy as the criterion for optimal hyperparameter value combinations.

For SVM, both *box constraint* and *kernel scale* were optimised. Box constraint search grid included 13 values in logarithmic space, with exponents from -2 to 4 and step of 0.5. These values were following:

[0.25 0.3536 0.5 0.7071 1 1.4142 2 2.8284 4 5.6569 8 11.3137 16]

Specification of the box constraint search grid followed the heuristic outlined in Hsu, Chang, & Lin (2003), but included a narrower range around the canonical value of 1 with an assumption that this may improve optimisation results.

Kernel scale search grid included 14 values, again calculated as powers of two with exponents from -2 to 4.5 with a step of 0.5. The search grid consisted of the following values:

[0.25 0.3536 0.5 0.7071 1 1.4142 2 2.8284 4 5.6569 8 11.3137 16 22.6274]

Specification of the kernel scale search grid again followed the heuristic from Hsu et al. (2003), but with a narrower range around the square roots of the number of features, with an assumption that this may improve optimisation results.

Ranges for both box constraint and kernel scale were deliberately constrained to decrease possibilities for overfitting. Overall, 182 (13 × 14) hyperparameter combinations were included in the SVM hyperparameter search grid.

For decision tree, only *minimum leaf size* was optimised as the most important classifier

hyperparameter (Mantovani et al., 2019). Larger minimum leaf sizes simultaneously constrain maximum number of decision tree splits, and some combinations of these two hyperparameters (e.g. low minimum leaf size and low maximum number of splits) can lead to less balanced trees which could be less generalisable. Maximum number of splits was therefore fixed and constrained by the sample size (N – 1). Search grid for the minimum leaf size followed the default MATLAB R2015b heuristic and included 10 values in logarithmic scaled space from two to half the sample size (log(2) to log(N/2)) with duplicates excluded. *Minimum parent size* was not optimised to reduce computation time, and also because it was shown previously that optimisation of this parameter is less effective compared to optimisation of the minimum leaf size (Mantovani et al., 2019).

In penalised logistic regression, *alpha* parameter controls the weight of L1 (lasso) versus L2 (ridge) regularisation. Alpha is a higher-level hyperparameter and was specified to the default value of 0.5, which equally balances ridge and lasso regularisation. The main optimised PLR hyperparameter was the *regularisation coefficient* (lambda). Search grid for lambda was specified following the heuristic implemented in MATLAB R2015b. The grid consisted of 20 values in a geometric sequence between the largest lambda value which results in a nonnull model (λ_{max}), and the value of $\frac{\lambda_{max}}{1000}$.

S1.3.4 Filter Feature Selection

The *p*-value threshold in the *t*-test filter was optimised through inner cross-validation. Search grid consisted of nine *p*-value thresholds between 0.01 and 0.05 with step of 0.005 and was the following:

[0.01 0.015 0.02 0.025 0.03 0.035 0.04 0.045 0.05]

Upper boundary in the above range was specified as the standard threshold for

statistically significant differences ($p \leq 0.05$, Bross, 1971). Lower boundary was specified as 0.01 because there were generally only few features which were significantly different between cases and controls at significance level $p \leq 0.01$ (uncorrected) across all datasets (Tables S1 – S10). During optimisation, filter threshold value with the highest inner cross-validation accuracy and lowest filtered number of features was selected for testing in outer cross-validation.

Classification analyses with filter feature selection was not performed for rMDD-STR sample with FA feature subset, because only one FA measure was significant at $p \leq 0.05$ (Table S7).

S1.3.5 Sequential Feature Elimination

In sequential feature elimination, inner cross-validation accuracy was used as the optimisation criterion. To enable reasonable computation times, sequential feature elimination was performed with elements of parallelisation as implemented in MATLAB R2015b. Each optimisation was performed on 8 cores of an Intel Xeon based computing cluster node with 2.4 GHz clock speed per core.

S1.3.6 Cross-validation Partitioning

Cross-validation was repeated 10 times with pre-determined random fold partitions in the smaller datasets (rMDD-STR and pMDD-UKB-ICD diagnostic criteria). This was not feasible for the larger datasets due to high computational complexity (cMDD-UKB and pMDD-UKB-CIDI diagnostic criteria). Cross-validation in the larger datasets was therefore performed only once for each classification method with the deterministically predefined fold partitions. Fold partitions for these datasets were defined separately for male and female cases and controls, with a greedy algorithm which aimed to maximally balance the folds with respect to age. The algorithm applied to define fold partitions was following:

1) Compute mean age for the sample and compute difference with mean age for each participant;
2) Sort participants in the order of increasing absolute difference with mean sample age;
3) Assign first ***k*** participants with smallest absolute difference with mean overall sample age to different folds, where ***k*** is the number of folds;
4) For each of the remaining ***N – k*** participants, assign participants to folds in the order of increasing participant age difference with the overall sample mean age; assign each participant ***p*** to the fold with minimal number of currently assigned participants, where the participant assignment results in the highest reduction of the difference between fold mean age and the overall sample mean age:
   - For each fold ***i*** with the minimal number of assigned participants compute difference between the fold mean age and the overall sample mean age (D_1^i);
   - For each fold ***i*** with the minimal number of assigned participants compute difference between the fold mean age and the overall sample mean age when participant ***p*** is added to the fold (D_2^i);
   - Assign participant ***p*** to the fold ***i*** with the highest value of $D_1^i - D_2^i$.

The folds were defined to be deterministically balanced with respect to age and sex with the above algorithm, and were thus non-random.

S1.3.7 Comparison of Classification Methods

For smaller datasets (rMDD-STR and pMDD-UKB-ICD diagnostic criteria) there were 100 accuracy estimates for each classification approach (10 cross-validation repetitions × 10 folds). The approaches were compared between each other using paired *t*-test with correction

for non-independence between accuracy estimates (Bouckaert & Frank, 2004; Nadeau & Bengio, 2003). Each classification approach was given a score according to the number of approaches which performed worse as assessed by the corrected paired *t*-test. For the larger datasets, repeated cross-validation was not feasible (cMDD-UKB and pMDD-UKB-CIDI diagnostic criteria), and hence classification approaches were compared using McNemar's test (McNemar, 1947). McNemar's tests were performed separately on the results from each cross-validation fold. Each approach was scored according to how many alternatives performed worse on each fold, and scores were then summed across the folds.

S2. RESULTS

S2.1 Brain Structure Differences

Correction for false discovery rate was performed separately for measures of cortical thickness, cortical surface areas, cortical or subcortical volumes, FA and MD.

Tables S1-S5 outline corrected and uncorrected significant ($p < 0.05$) case-control differences in brain morphometric measures in the five analysed samples. Where no differences where found for a sample, the related column in the table is omitted.

Tables S6-S10 outline corrected or uncorrected significant ($p < 0.05$) case-control differences in white matter integrity measures in the five samples. For white matter integrity, significant differences after FDR correction were only found in the three UK Biobank samples. Effects in light blue in Tables S8-S10 overlap between all three UKB samples, effects in light yellow overlap between cMDD-UKB and pMDD-UKB-CIDI, effects in light green overlap between pMDD-UKB-CIDI and pMDD-UKB-ICD samples.

S2.2 Classification Results

Results of cross-validation with sequential feature elimination with decision tree

classifier and combined brain morphometric feature set were not obtained for cMDD-UKB and pMDD-UKB-CIDI samples because each optimisation took longer than five days to run on 8 parallel cores of an Intel Xeon based computing cluster node (at 2.4 GHz clock speed per core). In addition, due to long optimisation times, classification analyses with decision tree classifier, sequential feature elimination and combined brain morphometric feature set were only performed once for rMDD-STR and pMDD-UKB-ICD samples, with predefined balanced fold partitions (section S1.3.6, no repeated cross-validation). This was also the case for decision tree classifier, sequential feature elimination and combined white-matter integrity feature set for pMDD-UKB-ICD sample.

For results of all classification analyses with brain morphometric and white-matter integrity features in cMDD-STR samples please see main text (Tables 4 and 5). Tables S11-S14 outline accuracies and ROC AUC measures for all classification attempts with brain morphometric features in rMDD-STR, cMDD-UKB, pMDD-UKB-CIDI and pMDD-UKB-ICD samples. Tables S15-S18 outline accuracies and ROC AUC measures for all classification attempts with white-matter integrity features in the four samples.

Classification analyses for cMDD-STR dataset with brain morphometric measures were repeated with a replaced set of control participants. The replaced controls were again matched to cases for age and sex (mean age 54.87, mean QIDS score 2.8), however matching for age was slightly worse compared to the original sample (Table 2 in the main text). Brief description of the main analysis results can be found in the results section of the main text. Table S19 outlines accuracies and ROC AUC measures for all classification attempts with the replaced set of controls in the cMDD-STR dataset with brain morphometric measures.

We additionally attempted classification with the two sets of control participants combined (original and replaced, twice as many controls compared to cases), with synthetic

minority oversampling to compensate for unbalanced class data (SMOTE, Chawla, Bowyer, Hall, & Kegelmeyer, 2002). Despite application of the SMOTE technique, this did not improve the original classification results and resulted in largely unbalanced sensitivities and specificities. Results for these classification attempts can be found in Table S20.

REFERENCES

Alfaro-Almagro, F., Jenkinson, M., Bangerter, N. K., Andersson, J. L. R., Griffanti, L., Douaud, G., … Smith, S. M. (2018). Image processing and Quality Control for the first 10,000 brain imaging datasets from UK Biobank. *NeuroImage*, *166*, 400–424.

American Psychiatric Association. (2000). *Diagnostic and Statistical Manual of Mental Disorders, Fourth Edition, Text Revision (DSM-IV-TR)* (4th ed., Vol. 1). Arlington, VA: American Psychiatric Association.

Arbabshirani, M. R., Plis, S., Sui, J., & Calhoun, V. D. (2017). Single subject prediction of brain disorders in neuroimaging: Promises and pitfalls. *NeuroImage*, *145*(Pt B), 137–165.

Bouckaert, R. R., & Frank, E. (2004). Evaluating the Replicability of Significance Tests for Comparing Learning Algorithms. In H. Dai, R. Srikant, & C. Zhang (Eds.), *Advances in Knowledge Discovery and Data Mining* (Vol. 3056, pp. 3–12). Berlin, Heidelberg: Springer Berlin Heidelberg.

Bross, I. J. D. (1971). Critical Levels, Statistical Language and Scientific Inference. In *Foundations of Statistical Inference*. Toronto: Holt, Rinehart & Winston of Canada, Ltd.

Chang, C.-C., & Lin, C.-J. (2011). LIBSVM: A library for support vector machines. *ACM Transactions on Intelligent Systems and Technology*, *2*(3), 1–27.

Chawla, N. V., Bowyer, K. W., Hall, L. O., & Kegelmeyer, W. P. (2002). SMOTE: Synthetic Minority Over-sampling Technique. *Journal of Artificial Intelligence Research*, *16*, 321–357.

Dadi, K., Rahim, M., Abraham, A., Chyzhyk, D., Milham, M., Thirion, B., … Alzheimer’s Disease Neuroimaging Initiative. (2019). Benchmarking functional connectome-based predictive models for resting-state fMRI. *NeuroImage*, *192*, 115–134.

Fernández-Delgado, M., Cernadas, E., Barro, S., & Amorim, D. (2014). Do we Need Hundreds of Classifiers to Solve Real World Classification Problems? *Journal of Machine Learning Research*, *15*(1), 3133–3181.

First, M., Gibbon, M., Spitzer, R., & Williams, J. (2002). Structured Clinical Interview for DSM-IV-TR Axis I Disorders (Research Version). New York State Psychiatric Institute.

Hsu, C.-W., Chang, C.-C., & Lin, C.-J. (2003). A Practical Guide to Support Vector Classification. Retrieved from http://www.csie.ntu.edu.tw/~cjlin/papers.html

Kambeitz, J., Cabral, C., Sacchet, M. D., Gotlib, I. H., Zahn, R., Serpa, M. H., … Koutsouleris, N. (2017). Detecting Neuroimaging Biomarkers for Depression: A Meta-analysis of Multivariate Pattern Recognition Studies. *Biological Psychiatry*, *82*(5), 330–338.

Kessler, R. C., Andrews, G., Mroczek, D., Ustun, B., & Wittchen, H.-U. (1998). The World Health Organization Composite International Diagnostic Interview short-form (CIDI-SF). *International Journal of Methods in Psychiatric Research*, *7*(4), 171–185.

Kroenke, K., & Spitzer, R. L. (2002). The PHQ-9: A New Depression Diagnostic and Severity Measure. *Psychiatric Annals*, *32*(9), 509–515.

Mantovani, R. G., Horváth, T., Cerri, R., Junior, S. B., Vanschoren, J., & de Carvalho, A. C. P. de L. F. (2019). An empirical study on hyperparameter tuning of decision trees. *ArXiv:1812.02207 [Cs, Stat]*. Retrieved from http://arxiv.org/abs/1812.02207

McNemar, Q. (1947). Note on the sampling error of the difference between correlated proportions or percentages. *Psychometrika*, *12*(2), 153–157.

Mori, S., & Crain, B. J. (2006). *MRI atlas of human white matter*. Amsterdam: Elsevier. Retrieved from http://search.ebscohost.com/login.aspx?direct=true&scope=site&db=nlebk&db=nlabk&AN=166331

Nadeau, C., & Bengio, Y. (2003). Inference for the Generalization Error. *Machine Learning*, *52*(3), 239–281.

Neilson, E., Shen, X., Cox, S. R., Clarke, T.-K., Wigmore, E. M., Gibson, J., … Lawrie, S. M. (2019). Impact of Polygenic Risk for Schizophrenia on Cortical Structure in UK Biobank. *Biological Psychiatry*, *86*(7), 536–544.

Pedregosa, F., Varoquaux, G., Gramfort, A., Michel, V., Thirion, B., Grisel, O., … Duchesnay, É. (2018). Scikit-learn: Machine Learning in Python. *ArXiv:1201.0490 [Cs]*. Retrieved from http://arxiv.org/abs/1201.0490

Smith, D. J., Nicholl, B. I., Cullen, B., Martin, D., Ul-Haq, Z., Evans, J., … Pell, J. P. (2013). Prevalence and characteristics of probable major depression and bipolar disorder within UK biobank: cross-sectional study of 172,751 participants. *PloS One*, *8*(11), e75362.

Smith, S., Alfaro-Almagro, F., & Miller, K. (2018). UK Biobank Brain Imaging Documentation. Oxford University. Retrieved from http://biobank.ndph.ox.ac.uk/showcase/docs/brain_mri.pdf

Smith, S., Jenkinson, M., Johansen-Berg, H., Rueckert, D., Nichols, T. E., Mackay, C. E., … Behrens, T. E. J. (2006). Tract-based spatial statistics: voxelwise analysis of multi-subject diffusion data. *NeuroImage*, *31*(4), 1487–1505.

Therneau, T., Atkinson, B., & Ripley, B. (2019). Rpart: Recursive Partitioning and Regression Trees. R Package Version 4.1-15. Retrieved from https://cran.r-project.org/web/packages/rpart/index.html

UK Biobank. (2014). Siemens Skyra Brain Scan Protocol. Retrieved from http://biobank.ndph.ox.ac.uk/showcase/docs/bmri_V4_23092014.pdf

UK Biobank. (2017, June 10). Mental health web-based questionnaire - Version 1.3. Retrieved from http://biobank.ndph.ox.ac.uk/showcase/docs/mental_health_online.pdf

World Health Organisation. (1992). ICD-10 Classifications of Mental and Behavioural Disorder: Clinical Descriptions and Diagnostic Guidelines. Geneva. World Health Organisation.

Yang, J., Zhang, M., Ahn, H., Zhang, Q., Jin, T. B., Li, I., … DeLorenzo, C. (2018). Development and evaluation of a multimodal marker of major depressive disorder. *Human Brain Mapping*, *39*(11), 4420–4439.

Zou, H., & Hastie, T. (2005). Regularization and variable selection via the elastic net. *Journal of the Royal Statistical Society: Series B (Statistical Methodology)*, *67*(2), 301–320.

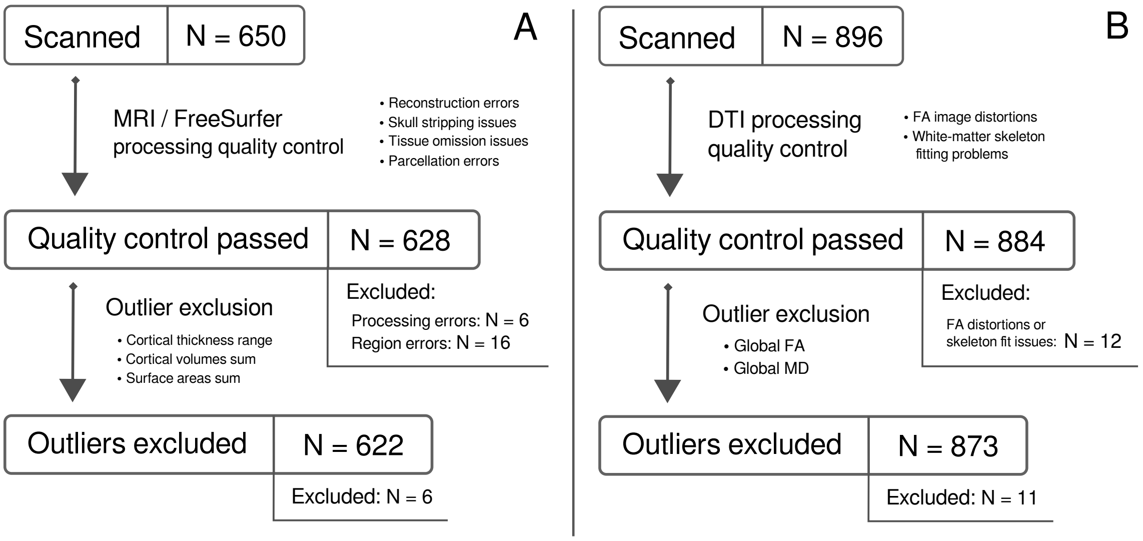

Figure S1. Flowcharts outlining participant exclusion in STRADL datasets of brain morphometric measures (A) and *white-matter integrity measures* (B).

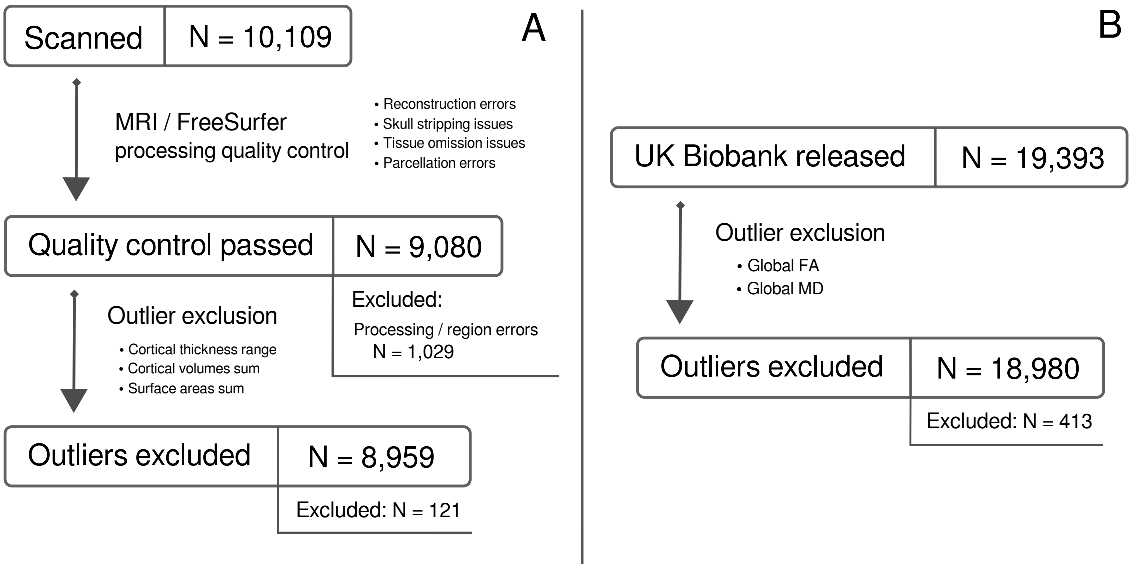

Figure S2. Flowcharts outlining participant exclusion in UK Biobank datasets of *brain morphometric measures* (A) and *white-matter integrity measures* (B).

Table S1

Brain morphometric measures with significant (uncorrected) differences between cases and controls in cMDD-STR sample (STRADL cohort)

Morphometric measure	*Brain region*	*Uncorrected P value*	*Effect size*
Cortical thickness	Rostral anterior cingulate (left)	0.0315	-0.5692
	Fusiform gyrus (right)	0.0121	-0.6693
	Inferior temporal gyrus (right)	0.0147	-0.6496
	Lateral orbitofrontal (right)	0.0048	-0.7570
Surface area	Superior frontal (left)	0.0467	0.5248
	Paracentral (right)	0.0300	0.5746
Volume	Caudal middle frontal (right)	0.0338	-0.5614
	Paracentral (right)	0.0202	0.6167

Note: No effects were significant after FDR correction.

Table S2

Brain morphometric measures with significant (uncorrected) differences between cases and controls in rMDD-STR sample (STRADL cohort)

Morphometric measure	*Brain region*	*Uncorrected P value*	*Effect size*
Cortical thickness	Pars orbitalis (right)	0.0469	0.2320
Surface area	Lingual gyrus (left)	0.0303	-0.2530
	Precentral (left)	0.0309	-0.2522
Volume	Precentral (left)	0.0378	-0.2426
	Brainstem	0.0174	-0.2781

Note: No effects were significant after FDR correction.

Table S3

Brain morphometric measures with significant (uncorrected) differences between cases and controls in cMDD-UKB sample (UK Biobank cohort)

Morphometric measure	*Brain region*	*Uncorrected P value*	*Effect size*
Cortical thickness	Banks of superior temporal sulcus (left)	0.0187	-0.1228
	Caudal anterior cingulate (left)	0.0308	-0.1128
	Pars opercularis (left)	0.0199	-0.1216
	Pars opercularis (right)	0.0371	-0.1089
	Posterior cingulate (left)	0.0499	-0.1024
	Precentral (left)	0.0090	-0.1365
	Precentral (right)	0.0292	-0.1139
	Superior frontal (left)	0.0021	-0.1604
	Superior frontal (right)	0.0164	-0.1253
	Superior temporal (left)	0.0395	-0.1075
	Insula (left)	0.0072	-0.1403
	Middle temporal (right)	0.0487	-0.1030
	Parahippocampal (right)	0.0279	-0.1148
Surface area	Caudal middle frontal (right)	0.0412	0.1066
	Precuneus (right)	0.0340	0.1107

Note: No effects were significant after FDR correction.

Table S4

Brain morphometric measures with significant differences between cases and controls in pMDD-UKB-CIDI sample (UK Biobank cohort)

Morphometric measure	*Brain region*	*Uncorrected P value*	*Corrected P value*	*Effect size*
Cortical thickness	Pars opercularis (left)	0.0454	n.s.	-0.0694
	Pars triangularis (left)	0.0025	n.s.	-0.1048
	Pars triangularis (right)	0.0181	n.s.	-0.0819
	Posterior cingulate (left)	0.0318	n.s.	-0.0744
	Rostral anterior cingulate (left)	0.0052	n.s.	-0.0969
	Lateral occipital (right)	0.0305	n.s.	0.0750
Surface area	Inferior temporal (left)	0.0049	n.s.	-0.0976
	Inferior temporal (right)	0.0254	n.s.	-0.0775
	Supramarginal (left)	0.0033	n.s.	-0.1019
Volume	Entorhinal (left)	0.0053	n.s.	-0.0966
	Inferior temporal (left)	0.0106	n.s.	-0.0886
	Inferior temporal (right)	0.0219	n.s.	-0.0795
	Supramarginal (left)	0.0001	0.0100	-0.1317
	Lateral orbitofrontal (right)	0.0459	n.s.	-0.0692
	Medial orbitofrontal (right)	0.0084	n.s.	-0.0914

Note: Significant effect after FDR correction is highlighted in light blue.

Table S5

Brain morphometric measures with significant differences between cases and controls in pMDD-UKB-ICD sample (UK Biobank cohort)

Morphometric measure	*Brain region*	*Uncorrected P value*	*Corrected P value*	*Effect size*
Cortical thickness	Caudal middle frontal (left)	0.0187	n.s.	-0.2828
	Fusiform gyrus (left)	0.0339	n.s.	-0.2547
	Pars opercularis (left)	0.0125	n.s.	-0.3005
	Pars triangularis (left)	0.0158	n.s.	-0.2903
	Pars triangularis (right)	0.0117	n.s.	-0.3034
	Rostral middle frontal (left)	0.0267	n.s.	-0.2663
	Rostral middle frontal (right)	0.0379	n.s.	-0.2494
	Superior temporal (left)	0.0481	n.s.	-0.2372
	Inferior temporal (right)	0.0431	n.s.	-0.2428
	Isthmus (right)	0.0012	n.s.	-0.3898
	Pars orbitalis (right)	0.0356	n.s.	-0.2524
	Posterior cingulate (right)	0.0489	n.s.	-0.2365
Surface area	Entorhinal (left)	0.0003	0.0171	-0.4433
	Supramarginal (right)	0.0489	n.s.	-0.2364
	Frontal pole (right)	0.0258	n.s.	0.2679
Volume	Cuneus (left)	0.0286	n.s.	0.2631
	Entorhinal (left)	0.0213	n.s.	-0.2768
	Fusiform gyrus (left)	0.0228	n.s.	-0.2737
	Inferior temporal (right)	0.0455	n.s.	-0.2402
	Pars triangularis (right)	0.0163	n.s.	-0.2889
	Cerebellar grey matter (left)	0.0456	n.s.	-0.2400
	Cerebellar white matter (left)	0.0166	n.s.	-0.2879
	Cerebellar white matter (right)	0.0135	n.s.	-0.2973

Note: Significant effect after FDR correction is highlighted in light blue.

Table S6

White-matter integrity measures with significant (uncorrected) differences between cases and controls in cMDD-STR sample (STRADL cohort)

Integrity measure	*White-matter tract*	*Uncorrected P value*	*Effect size*
FA	Anterior limb of internal capsule (left)	0.0284	-0.4994
	External capsule (right)	0.0464	-0.4527
	Splenium of corpus callosum	0.0496	-0.4459
	Superior frontooccipital fasciculus (left)	0.0381	-0.4717
	Superior frontooccipital fasciculus (right)	0.0354	-0.4787
MD	Cingulum cingulate gyrus (right)	0.0435	-0.4588
	Superior frontooccipital fasciculus (right)	0.0493	0.4467
	Uncinate fasciculus (right)	0.0273	0.5029

Note: No effects were significant after FDR correction.

Table S7

White-matter integrity measures with significant (uncorrected) differences between cases and controls in rMDD-STR sample (STRADL cohort)

Integrity measure	*White-matter tract*	*Uncorrected P value*	*Effect size*
FA	Inferior frontooccipital fasciculus (left)	0.0462	-0.1990
MD	External capsule (left)	0.0040	0.2880
	Sagittal stratum (right)	0.0273	-0.2205

Note: No effects were significant after FDR correction.

Table S8

White-matter integrity measures with significant (corrected) differences between cases and controls in cMDD-UKB sample (UK Biobank cohort)

Integrity measure	*White-matter tract*	*Uncorrected P value*	*Corrected P value*	*Effect size*
FA	Cingulum hippocampus (right)	0.0004	0.0182	0.1329
MD	Anterior limb of internal capsule (right)	0.0007	0.0178	0.1261
	Anterior limb of internal capsule (left)	0.0026	0.0252	0.1124
	Superior corona radiata (right)	0.0021	0.0248	0.1151
	Superior corona radiata (left)	0.0019	0.0248	0.1161
	Superior frontooccipital fasciculus (right)	0.0007	0.0178	0.1264

Note: Effects in yellow overlap with pMDD-UKB-CIDI sample. Effects in blue overlap with both pMDD-UKB-CIDI and pMDD-UKB-ICD samples. Effect sizes are in Cohen's *d* values.

Table S9

White-matter integrity measures with significant (corrected) differences between cases and controls in pMDD-UKB-CIDI sample (UK Biobank cohort)

Integrity measure	*White-matter tract*	*Uncorrected P value*	*Corrected P value*	*Effect size*
FA	Genu of corpus callosum	0.0010	0.0067	-0.0796
	Fornix	0.0081	0.0228	-0.0641
	Inferior cerebellar peduncle (right)	0.0041	0.0157	-0.0695
	Inferior cerebellar peduncle (left)	0.0001	0.0014	-0.0933
	Superior cerebellar peduncle (right)	0.0049	0.0157	-0.0681
	Superior cerebellar peduncle (left)	0.0048	0.0157	-0.0683
	Anterior limb of internal capsule (right)	0.0041	0.0157	-0.0694
	Anterior limb of internal capsule (left)	0.0008	0.0064	-0.0811
	Anterior corona radiata (right)	0.0048	0.0157	-0.0683
	Anterior corona radiata (left)	0.0101	0.0271	-0.0622
	Superior corona radiata (left)	0.0039	0.0157	-0.0698
	Posterior thalamic radiation (right)	0.00009	0.0014	-0.0946
	Posterior thalamic radiation (left)	0.0002	0.0023	-0.0888
	Sagittal stratum (left)	0.0058	0.0173	-0.0668
	Fornix (cres) / Stria terminalis (right)	0.0030	0.0157	-0.0717
	Fornix (cres) / Stria terminalis (left)	0.0011	0.0067	-0.0788
	Superior frontooccipital fasciculus (right)	0.00005	0.0012	-0.0983
	Superior frontooccipital fasciculus (left)	0.00004	0.0012	-0.0988
MD	Pontine crossing tract	0.0054	0.0377	-0.0673
	Genu of corpus callosum	0.0079	0.0469	0.0644
	Corticospinal tract (left)	0.0031	0.0300	-0.0715
	Anterior limb of internal capsule (right)	0.0103	0.0492	0.0621
	Anterior limb of internal capsule (left)	0.0095	0.0492	0.0627
	Superior corona radiata (right)	0.0003	0.0065	0.0881
	Superior corona radiata (left)	0.00005	0.0023	0.0984
	Fornix (cres) / Stria terminalis (right)	0.0050	0.0372	0.0679
	Superior frontooccipital fasciculus (right)	0.0020	0.0236	0.0749
	Superior frontooccipital fasciculus (left)	0.0008	0.0126	0.0813

Note: Effects in yellow overlap with cMDD-UKB sample. Effects in green overlap with pMDD-UKB-ICD sample. Effects in blue overlap with both cMDD-UKB and pMDD-UKB-ICD samples. Effect sizes are in Cohen's *d* values.

Table S10

White-matter integrity measures with significant (corrected) differences between cases and controls in pMDD-UKB-ICD sample (UK Biobank cohort)

Integrity measure	*White-matter tract*	*Uncorrected P value*	*Corrected P value*	*Effect size*
FA	Genu of corpus callosum	0.0003	0.0083	-0.3031
	Body of corpus callosum	0.0013	0.0192	-0.2685
	Superior cerebellar peduncle (right)	0.0048	0.0289	-0.2355
	Anterior limb of internal capsule (right)	0.0016	0.0192	-0.2638
	Anterior limb of internal capsule (left)	0.0082	0.0392	-0.2208
	Anterior corona radiata (right)	0.0026	0.0204	-0.2521
	Posterior corona radiata (left)	0.0030	0.0205	-0.2481
	Posterior thalamic radiation (right)	0.0003	0.0083	-0.2994
	Posterior thalamic radiation (left)	0.0023	0.0204	-0.2543
	Fornix (cres) / Stria terminalis (left)	0.0060	0.0316	-0.2298
MD	Genu of corpus callosum	0.0002	0.0094	0.3068
	Body of corpus callosum	0.0004	0.0094	0.2968
	Superior corona radiata (right)	0.0041	0.0479	0.2399
	Superior corona radiata (left)	0.0010	0.0162	0.2749
	Cingulum cingulate gyrus (right)	0.0050	0.0479	0.2345

Note: Effects in green overlap with pMDD-UKB-CIDI sample. Effects in blue overlap with both cMDD-UKB and pMDD-UKB-CIDI samples. Effect sizes are in Cohen's *d* values.

Table S11

Case-control classification accuracies and ROC AUC measures (on repeated cross-validation) with *brain morphometric* features in rMDD-STR sample (148 cases and 148 controls, STRADL cohort)

Classif. type	*Feature selection*	*Hyperparam. optimisation*	*Outer CV*	*Inner CV*	*Feature domain*	*Classification accuracy (sensitivity / specificity)*	*ROC AUC*	*Score*
PLR	Embedded	Grid search	10-fold	10-fold	Thickness	50.60% (49.73% / 51.47%)	0.511	0
					Surface area	49.95% (52.79% / 47.18%)	0.500	0
					Volume	51.68% (54.41% / 48.95%)	0.502	0
					Subcortical	54.66% (56.76% / 52.59%)	0.578	2
					Combined	50.01% (51.64% / 48.38%)	0.495	0
SVM	None	None	10-fold	-	Thickness	51.07% (51.10% / 51.04%)	0.540	0
					Surface area	49.87% (50.61% / 49.12%)	0.509	0
					Volume	54.51% (55.63% / 53.33%)	0.556	1
					Subcortical	55.04% (54.89% / 55.19%)	0.557	3
					Combined	51.84% (50.37% / 53.29%)	0.540	0
		Grid search		10-fold	Thickness	52.76% (58.38% / 47.18%)	0.521	0
					Surface area	49.75% (43.32% / 56.35%)	0.500	0
					Volume	52.60% (54.60% / 50.65%)	0.518	0
					Subcortical	50.54% (46.72% / 54.42%)	0.525	0
					Combined	51.99% (52.18% / 51.89%)	0.509	0
	Statistical filter	None			Combined	51.39% (57.81% / 44.92%)	0.524	0
		Grid search			Combined	52.15% (54.63% / 49.67%)	0.524	0
	Sequential elimination	None			Thickness	50.52% (49.49% / 51.59%)	0.530	0
					Surface area	49.64% (52.36% / 46.88%)	0.498	0
					Volume	54.64% (55.10% / 54.16%)	0.550	1
					Subcortical	53.76% (51.17% / 56.36%)	0.558	0
					Combined	52.89% (49.57% / 56.21%)	0.542	0
DT	None	None	10-fold	-	Thickness	47.77% (48.06% / 47.42%)	0.469	0
					Surface area	51.63% (48.14% / 55.04%)	0.520	0
					Volume	50.04% (48.24% / 51.87%)	0.510	0
					Subcortical	52.59% (50.40% / 54.76%)	0.553	0
					Combined	57.09% (56.64% / 57.47%)	0.591	11
		Grid search		10-fold	Thickness	47.07% (45.84% / 48.24%)	0.472	0
					Surface area	55.63% (57.50% / 53.76%)	0.561	5
					Volume	49.30% (49.80% / 48.77%)	0.502	0
					Subcortical	51.66% (53.30% / 50.00%)	0.525	0
					Combined	55.04% (55.05% / 54.96%)	0.564	1
	Statistical filter	None			Combined	52.79% (51.62% / 53.98%)	0.542	0
		Grid search			Combined	57.48% (52.57% / 62.35%)	0.572	13
	Sequential elimination	None			Thickness	48.55% (48.95% / 48.11%)	0.475	0
					Surface area	52.49% (50.11% / 54.76%)	0.530	0
					Volume	48.95% (47.30% / 50.52%)	0.496	0
					Subcortical	52.13% (49.76% / 54.54%)	0.551	0
					Combined	53.42% (48.79% / 58.07%)	0.532	-

Note: Top accuracies for SVM, PLR and DT classifiers, and score for the best overall approach are highlighted in light blue. Optimisation with DT classifier, sequential feature elimination and combined feature set was only performed once (no repetitions), hence score not shown (section S2.2).

Table S12

Case-control classification accuracies and ROC AUC measures (on single cross-validation) with *brain morphometric* features in cMDD-UKB sample (735 cases and 735 controls, UK Biobank cohort)

Classif. type	*Feature selection*	*Hyperparam. optimisation*	*Outer CV*	*Inner CV*	*Feature domain*	*Classification accuracy (sensitivity / specificity)*	*ROC AUC*	*Score*
PLR	Embedded	Grid search	10-fold	10-fold	Thickness	52.80% (52.66% / 52.92%)	0.540	58
					Surface area	50.40% (50.21% / 50.61%)	0.488	11
					Volume	50.06% (50.62% / 49.51%)	0.505	9
					Subcortical	50.27% (53.64% / 46.88%)	0.505	5
					Combined	52.31% (51.99% / 52.64%)	0.519	30
SVM	None	None	10-fold	-	Thickness	50.95% (51.56% / 50.32%)	0.517	24
					Surface area	50.41% (51.16% / 49.67%)	0.502	9
					Volume	51.63% (52.52% / 50.76%)	0.516	18
					Subcortical	49.94% (47.21% / 52.64%)	0.506	18
					Combined	51.22% (51.42% / 51.01%)	0.524	15
		Grid search		10-fold	Thickness	51.09% (48.84% / 53.34%)	0.519	19
					Surface area	49.87% (44.88% / 54.81%)	0.498	6
					Volume	49.86% (50.89% / 48.89%)	0.497	3
					Subcortical	50.35% (46.00% / 54.69%)	0.484	30
					Combined	50.75% (52.65% / 48.85%)	0.515	17
	Statistical filter	None		10-fold	Combined	49.18% (52.25% / 46.12%)	0.501	9
		Grid search			Combined	48.70% (51.56% / 45.86%)	0.482	16
	Sequential elimination	None			Thickness	51.43% (51.16% / 51.67%)	0.520	34
					Surface area	50.00% (50.34% / 49.66%)	0.495	10
					Volume	51.09% (51.70% / 50.50%)	0.505	14
					Subcortical	49.32% (44.75% / 53.88%)	0.495	3
					Combined	52.11% (51.97% / 52.25%)	0.527	30
DT	None	None	10-fold	-	Thickness	48.98% (50.60% / 47.32%)	0.495	25
					Surface area	50.06% (49.64% / 50.46%)	0.493	12
					Volume	50.82% (44.95% / 56.78%)	0.519	27
					Subcortical	49.73% (65.64% / 33.86%)	0.506	6
					Combined	49.12% (46.91% / 51.28%)	0.488	3
		Grid search		10-fold	Thickness	51.56% (53.99% / 49.09%)	0.513	16
					Surface area	48.85% (53.44% / 44.17%)	0.484	17
					Volume	51.76% (55.63% / 47.87%)	0.521	24
					Subcortical	47.01% (48.82% / 45.17%)	0.465	0
					Combined	51.16% (50.08% / 52.25%)	0.495	15
	Statistical filter	None		10-fold	Combined	47.89% (48.40% / 47.36%)	0.478	1
		Grid search			Combined	49.38% (48.31% / 50.46%)	0.479	9
	Sequential elimination	None			Thickness	49.46% (46.93% / 52.00%)	0.491	9
					Surface area	51.30% (55.50% / 47.10%)	0.508	34
					Volume	50.28% (50.62% / 49.92%)	0.499	18
					Subcortical	49.79% (51.63% / 47.91%)	0.500	4
					Combined	N/A	N/A	-

Note: Top accuracies for SVM, PLR and DT classifiers, and score for the best overall approach are highlighted in light blue. Optimisation with sequential feature elimination for decision tree and combined feature set was not performed due to high computational complexity (section S2.2).

Table S13

Case-control classification accuracies and ROC AUC measures (on single cross-validation) with *brain morphometric* features in pMDD-UKB-CIDI sample (1665 cases and 1665 controls, UK Biobank cohort)

Classif. type	*Feature selection*	*Hyperparam. optimisation*	*Outer CV*	*Inner CV*	*Feature domain*	*Classification accuracy (sensitivity / specificity)*	*ROC AUC*	*Score*
PLR	Embedded	Grid search	10-fold	10-fold	Thickness	52.07% (53.00% / 51.14%)	0.534	24
					Surface area	51.41% (52.94% / 49.88%)	0.525	9
					Volume	52.79% (53.54% / 52.04%)	0.535	37
					Subcortical	48.20% (59.91% / 36.52%)	0.477	2
					Combined	52.94% (53.60% / 52.28%)	0.543	30
SVM	None	None	10-fold	-	Thickness	53.63% (53.72% / 53.54%)	0.532	64
					Surface area	49.40% (48.68% / 50.12%)	0.499	5
					Volume	52.01% (51.74% / 52.29%)	0.531	28
					Subcortical	51.08% (51.79% / 50.37%)	0.517	18
					Combined	51.77% (52.64% / 50.90%)	0.528	16
		Grid search		10-fold	Thickness	53.00% (52.70% / 53.30%)	0.533	48
					Surface area	50.33% (54.79% / 45.88%)	0.502	8
					Volume	53.09% (54.26% / 51.93%)	0.533	42
					Subcortical	51.53% (51.98% / 51.08%)	0.512	8
					Combined	51.41% (51.19% / 51.63%)	0.528	15
	Statistical filter	None			Combined	52.85% (58.16% / 47.54%)	0.547	39
		Grid search			Combined	52.46% (56.36% / 48.56%)	0.546	26
	Sequential elimination	None			Thickness	52.61% (52.34% / 52.88%)	0.527	32
					Surface area	49.01% (49.10% / 48.92%)	0.495	3
					Volume	52.43% (52.94% / 51.93%)	0.532	22
					Subcortical	49.37% (53.12% / 45.62%)	0.500	3
					Combined	51.53% (52.52% / 50.54%)	0.528	15
DT	None	None	10-fold	-	Thickness	49.85% (39.57% / 60.11%)	0.500	2
					Surface area	51.59% (64.88% / 38.32%)	0.517	18
					Volume	50.66% (60.67% / 40.64%)	0.516	12
					Subcortical	51.65% (71.29% / 32.01%)	0.509	11
					Combined	51.08% (61.22% / 40.94%)	0.524	13
		Grid search		10-fold	Thickness	49.10% (52.89% / 45.31%)	0.492	1
					Surface area	50.45% (46.83% / 54.07%)	0.503	5
					Volume	51.98% (53.89% / 50.07%)	0.526	21
					Subcortical	49.61% (48.26% / 50.96%)	0.500	4
					Combined	50.75% (45.96% / 55.52%)	0.517	10
	Statistical filter	None			Combined	52.67% (66.87% / 38.49%)	0.522	18
		Grid search			Combined	50.03% (46.64% / 53.42%)	0.511	6
	Sequential elimination	None			Thickness	50.60% (48.53% / 52.69%)	0.512	6
					Surface area	51.62% (68.99% / 34.25%)	0.513	13
					Volume	49.76% (63.51% / 36.02%)	0.505	3
					Subcortical	51.44% (75.75% / 27.13%)	0.518	8
					Combined	N/A	N/A	-

Note: Top accuracies for SVM, PLR and DT classifiers, and score for the best overall approach are highlighted in light blue. Optimisation with sequential feature elimination for decision tree and combined feature set was not performed due to high computational complexity (section S2.2).

Table S14

Case-control classification accuracies and ROC AUC measures (on repeated cross-validation) with *brain morphometric* features in pMDD-UKB-ICD sample (140 cases and 140 controls, UK Biobank cohort)

Classif. type	*Feature selection*	*Hyperparam. optimisation*	*Outer CV*	*Inner CV*	*Feature domain*	*Classification accuracy (sensitivity / specificity)*	*ROC AUC*	*Score*
PLR	Embedded	Grid search	10-fold	10-fold	Thickness	52.68% (53.43% / 51.93%)	0.547	0
PLR	Embedded	Grid search	10-fold	10-fold	Surface area	54.39% (53.36% / 55.43%)	0.584	0
PLR	Embedded	Grid search	10-fold	10-fold	Volume	52.36% (53.00% / 51.71%)	0.539	0
PLR	Embedded	Grid search	10-fold	10-fold	Subcortical	53.57% (53.93% / 53.21%)	0.541	0
PLR	Embedded	Grid search	10-fold	10-fold	Combined	60.29% (61.86% / 58.71%)	0.645	20
SVM	None	None	10-fold	-	Thickness	56.14% (64.79% / 47.50%)	0.576	0
SVM	None	None	10-fold	-	Surface area	50.71% (51.21% / 50.21%)	0.530	0
SVM	None	None	10-fold	-	Volume	53.75% (53.93% / 53.57%)	0.553	0
SVM	None	None	10-fold	-	Subcortical	51.75% (41.79% / 61.71%)	0.506	0
SVM	None	None	10-fold	-	Combined	55.11% (54.21% / 56.00%)	0.592	0
SVM	None	Grid search	10-fold	10-fold	Thickness	54.46% (61.93% / 47.00%)	0.550	0
SVM	None	Grid search	10-fold	10-fold	Surface area	51.93% (51.86% / 52.00%)	0.552	0
SVM	None	Grid search	10-fold	10-fold	Volume	54.21% (52.86% / 55.57%)	0.560	0
SVM	None	Grid search	10-fold	10-fold	Subcortical	51.82% (42.14% / 61.50%)	0.526	0
SVM	None	Grid search	10-fold	10-fold	Combined	54.89% (53.21% / 56.57%)	0.585	0
SVM	Statistical filter	None	10-fold	10-fold	Combined	57.21% (57.00% / 57.43%)	0.599	4
SVM	Statistical filter	Grid search	10-fold	10-fold	Combined	58.46% (59.07% / 57.86%)	0.608	9
SVM	Sequential elimination	None	10-fold	10-fold	Thickness	56.71% (64.29% / 49.14%)	0.588	0
SVM	Sequential elimination	None	10-fold	10-fold	Surface area	51.82% (52.86% / 50.79%)	0.538	0
SVM	Sequential elimination	None	10-fold	10-fold	Volume	53.29% (52.79% / 53.79%)	0.546	0
SVM	Sequential elimination	None	10-fold	10-fold	Subcortical	52.32% (41.50% / 63.14%)	0.511	0
SVM	Sequential elimination	None	10-fold	10-fold	Combined	57.64% (59.00% / 56.29%)	0.613	6
DT	None	None	10-fold	-	Thickness	55.46% (56.64% / 54.29%)	0.562	0
DT	None	None	10-fold	-	Surface area	58.50% (60.64% / 56.36%)	0.611	8
DT	None	None	10-fold	-	Volume	49.93% (49.79% / 50.07%)	0.495	0
DT	None	None	10-fold	-	Subcortical	50.54% (45.29% / 55.79%)	0.501	0
DT	None	None	10-fold	-	Combined	51.82% (52.71% / 50.93%)	0.543	0
DT	None	Grid search	10-fold	10-fold	Thickness	54.07% (55.07% / 53.07%)	0.555	0
DT	None	Grid search	10-fold	10-fold	Surface area	54.64% (55.43% / 53.86%)	0.569	0
DT	None	Grid search	10-fold	10-fold	Volume	50.29% (48.14% / 52.43%)	0.498	0
DT	None	Grid search	10-fold	10-fold	Subcortical	50.68% (47.50% / 53.86%)	0.516	0
DT	None	Grid search	10-fold	10-fold	Combined	51.54% (51.71% / 51.36%)	0.509	0
DT	Statistical filter	None	10-fold	10-fold	Combined	54.64% (57.50% / 51.79%)	0.554	0
DT	Statistical filter	Grid search	10-fold	10-fold	Combined	52.86% (53.50% / 52.21%)	0.526	0
DT	Sequential elimination	None	10-fold	10-fold	Thickness	56.00% (58.79% / 53.21%)	0.569	0
DT	Sequential elimination	None	10-fold	10-fold	Surface area	55.61% (58.43% / 52.79%)	0.591	0
DT	Sequential elimination	None	10-fold	10-fold	Volume	49.36% (47.50% / 51.21%)	0.495	0
DT	Sequential elimination	None	10-fold	10-fold	Subcortical	51.11% (43.71% / 58.50%)	0.502	0
DT	Sequential elimination	None	10-fold	10-fold	Combined	48.95% (47.91% / 49.75%)	0.492	-

Note: Top accuracies for SVM, PLR and DT classifiers, and score for the best overall approach are highlighted in light blue. Optimisation with DT classifier, sequential feature elimination and combined feature set was only performed once (no repetitions), hence score not shown (section S2.2).

Table S15

Case-control classification accuracies and ROC AUC measures (on repeated cross-validation) with *white-matter integrity* features in rMDD-STR sample (202 cases and 202 controls, STRADL cohort)

Classif. type	*Feature selection*	*Hyperparam. optimisation*	*Outer CV*	*Inner CV*	*Feature domain*	*Classification accuracy (sensitivity / specificity)*	*ROC AUC*	*Score*
PLR	Embedded	Grid search	10-fold	10-fold	FA	46.28% (50.39% / 42.18%)	0.445	0
					MD	55.15% (53.48% / 56.78%)	0.560	7
					Combined	52.20% (51.24% / 53.16%)	0.542	1
SVM	None	None	10-fold	-	FA	47.17% (50.86% / 43.49%)	0.464	0
					MD	55.08% (54.23% / 55.90%)	0.569	6
					Combined	54.61% (55.61% / 53.61%)	0.544	5
		Grid search		10-fold	FA	46.63% (52.75% / 40.70%)	0.471	0
					MD	55.54% (59.16% / 51.92%)	0.560	6
					Combined	52.86% (55.83% / 49.92%)	0.526	4
	Statistical filter	None			MD	53.04% (43.41% / 62.62%)	0.557	4
					Combined	50.63% (39.91% / 61.35%)	0.528	0
		Grid search			MD	53.24% (51.72% / 54.76%)	0.550	4
					Combined	51.59% (49.20% / 54.05%)	0.523	1
	Sequential elimination	None			FA	47.12% (50.50% / 43.71%)	0.465	0
					MD	54.13% (52.89% / 55.35%)	0.559	4
					Combined	53.74% (54.01% / 53.47%)	0.541	4
DT	None	None	10-fold	-	FA	48.69% (46.40% / 50.98%)	0.485	0
					MD	53.60% (51.18% / 55.91%)	0.540	4
					Combined	51.40% (46.94% / 55.87%)	0.511	0
		Grid search		10-fold	FA	54.55% (66.58% / 42.53%)	0.544	4
					MD	52.82% (53.37% / 52.26%)	0.536	3
					Combined	51.80% (56.69% / 46.91%)	0.513	1
	Statistical filter	None			MD	52.89% (55.77% / 50.07%)	0.545	4
					Combined	52.59% (55.64% / 49.56%)	0.539	3
		Grid search			MD	54.37% (56.59% / 52.15%)	0.562	6
					Combined	53.25% (54.23% / 52.23%)	0.544	4
	Sequential elimination	None			FA	48.85% (43.87% / 53.74%)	0.486	0
					MD	53.09% (50.58% / 55.59%)	0.540	4
					Combined	49.86% (45.64% / 54.08%)	0.508	0

Note: Top accuracies for SVM, PLR and DT classifiers, and score for the best overall approach are highlighted in light blue. Optimisation with filter feature selection with FA features was not performed because there was only one feature significantly different between cases and controls at $p < 0.05$ uncorrected (Table S8).

Table S16

Case-control classification accuracies and ROC AUC measures (on single cross-validation) with *white-matter integrity* features in cMDD-UKB sample (1435 cases and 1435 controls, UK Biobank cohort)

Classif. type	*Feature selection*	*Hyperparam. optimisation*	*Outer CV*	*Inner CV*	*Feature domain*	*Classification accuracy (sensitivity / specificity)*	*ROC AUC*	*Score*
PLR	Embedded	Grid search	10-fold	10-fold	FA	53.07% (53.45% / 52.69%)	0.536	28
					MD	50.63% (50.67% / 50.59%)	0.519	9
					Combined	53.63% (52.90% / 54.36%)	0.548	55
SVM	None	None	10-fold	-	FA	53.24% (53.03% / 53.45%)	0.537	23
					MD	53.24% (51.91% / 54.56%)	0.540	23
					Combined	52.33% (49.96% / 54.70%)	0.540	26
		Grid search		10-fold	FA	52.68% (50.66% / 54.71%)	0.539	24
					MD	51.84% (50.46% / 53.24%)	0.524	13
					Combined	53.69% (52.96% / 54.42%)	0.543	37
	Statistical filter	None			FA	53.21% (48.71% / 57.71%)	0.544	24
					MD	51.19% (34.70% / 67.68%)	0.508	5
					Combined	52.54% (43.76% / 61.33%)	0.531	16
		Grid search			FA	51.81% (48.09% / 55.54%)	0.537	9
					MD	51.67% (42.38% / 60.98%)	0.520	23
					Combined	51.88% (47.88% / 55.88%)	0.521	20
	Sequential elimination	None			FA	52.44% (51.98% / 52.90%)	0.537	15
					MD	52.12% (50.80% / 53.45%)	0.534	17
					Combined	53.73% (51.08% / 56.37%)	0.549	39
DT	None	None	10-fold	-	FA	50.45% (44.36% / 56.52%)	0.512	0
					MD	50.35% (40.15% / 60.58%)	0.513	1
					Combined	51.88% (43.28% / 60.48%)	0.523	7
		Grid search		10-fold	FA	52.09% (54.45% / 49.76%)	0.520	28
					MD	51.99% (51.06% / 52.92%)	0.515	30
					Combined	51.12% (52.82% / 49.42%)	0.514	3
	Statistical filter	None			FA	52.12% (48.76% / 55.46%)	0.533	14
					MD	51.39% (37.92% / 64.86%)	0.513	2
					Combined	50.35% (39.01% / 61.67%)	0.503	0
		Grid search			FA	53.17% (54.86% / 51.50%)	0.532	27
					MD	50.69% (53.29% / 48.07%)	0.514	8
					Combined	51.15% (52.34% / 49.95%)	0.510	5
	Sequential elimination	None			FA	50.39% (43.03% / 57.71%)	0.501	14
					MD	49.79% (44.04% / 55.57%)	0.503	7
					Combined	52.61% (58.74% / 46.49%)	0.527	14

Note: Top accuracies for SVM, PLR and DT classifiers, and score for the best overall approach are highlighted in light blue.

Table S17

Case-control classification accuracies and ROC AUC measures (on single cross-validation) with *white-matter integrity* features in pMDD-UKB-CIDI sample (3418 cases and 3418 controls, UK Biobank cohort)

Classif. type	*Feature selection*	*Hyperparam. optimisation*	*Outer CV*	*Inner CV*	*Feature domain*	*Classification accuracy (sensitivity / specificity)*	*ROC AUC*	*Score*
PLR	Embedded	Grid search	10-fold	10-fold	FA	52.18% (51.14% / 53.22%)	0.524	16
					MD	51.95% (50.26% / 53.63%)	0.529	5
					Combined	52.22% (51.14% / 53.31%)	0.532	12
SVM	None	None	10-fold	-	FA	51.07% (50.41% / 51.73%)	0.514	3
					MD	51.70% (48.24% / 55.15%)	0.518	6
					Combined	52.25% (53.13% / 51.38%)	0.527	19
		Grid search		10-fold	FA	52.22% (50.20% / 54.24%)	0.529	13
					MD	51.52% (45.05% / 57.99%)	0.523	7
					Combined	51.87% (50.90% / 52.84%)	0.530	12
	Statistical filter	None			FA	52.02% (47.48% / 56.55%)	0.525	10
					MD	52.12% (41.05% / 63.19%)	0.526	12
					Combined	52.22% (45.70% / 58.75%)	0.528	16
		Grid search			FA	51.62% (51.76% / 51.49%)	0.525	11
					MD	51.64% (44.61% / 58.66%)	0.524	6
					Combined	51.86% (47.25% / 56.46%)	0.529	8
	Sequential elimination	None			FA	51.84% (50.35% / 53.34%)	0.522	6
					MD	51.05% (47.42% / 54.68%)	0.517	7
					Combined	52.68% (53.63% / 51.73%)	0.531	35
DT	None	None	10-fold	-	FA	49.18% (46.39% / 51.97%)	0.499	0
					MD	50.66% (33.41% / 67.90%)	0.505	1
					Combined	51.51% (44.71% / 58.30%)	0.517	2
		Grid search		10-fold	FA	51.17% (57.00% / 45.34%)	0.509	5
					MD	50.41% (60.63% / 40.19%)	0.510	0
					Combined	50.15% (50.34% / 49.96%)	0.511	0
	Statistical filter	None			FA	50.41% (40.79% / 60.03%)	0.509	4
					MD	52.17% (39.29% / 65.04%)	0.523	11
					Combined	51.13% (50.85% / 51.40%)	0.517	8
		Grid search			FA	50.44% (55.25% / 45.63%)	0.501	4
					MD	51.87% (54.21% / 49.53%)	0.521	11
					Combined	51.48% (48.65% / 54.30%)	0.519	5
	Sequential elimination	None			FA	49.96% (50.55% / 49.36%)	0.497	0
					MD	51.00% (42.37% / 59.62%)	0.508	2
					Combined	51.23% (44.94% / 57.51%)	0.510	4

Note: Top accuracies for SVM, PLR and DT classifiers, and score for the best overall approach are highlighted in light blue.

Table S18

Case-control classification accuracies and ROC AUC measures (on repeated cross-validation) with *white-matter integrity* features in pMDD-UKB-ICD sample (289 cases and 289 controls, UK Biobank cohort)

Classif. type	*Feature selection*	*Hyperparam. optimisation*	*Outer CV*	*Inner CV*	*Feature domain*	*Classification accuracy (sensitivity / specificity)*	*ROC AUC*	*Score*
PLR	Embedded	Grid search	10-fold	10-fold	FA	54.61% (52.98% / 56.25%)	0.555	5
					MD	54.21% (53.22% / 55.22%)	0.555	1
					Combined	53.27% (51.69% / 54.86%)	0.547	1
SVM	None	None	10-fold	-	FA	54.34% (54.08% / 54.61%)	0.550	1
					MD	54.98% (56.61% / 53.37%)	0.578	6
					Combined	55.80% (56.41% / 55.21%)	0.579	11
		Grid search		10-fold	FA	53.72% (57.52% / 49.95%)	0.558	1
					MD	54.05% (63.04% / 45.06%)	0.551	3
					Combined	55.56% (61.29% / 49.85%)	0.565	9
	Statistical filter	None			FA	55.97% (57.20% / 54.77%)	0.569	11
					MD	56.18% (68.56% / 43.83%)	0.566	12
					Combined	55.73% (61.31% / 50.16%)	0.566	11
		Grid search			FA	53.42% (56.88% / 49.97%)	0.548	1
					MD	54.67% (64.04% / 45.31%)	0.563	1
					Combined	54.31% (56.33% / 52.31%)	0.556	2
	Sequential elimination	None			FA	54.63% (54.14% / 55.13%)	0.555	2
					MD	54.74% (55.99% / 53.52%)	0.573	3
					Combined	55.97% (56.51% / 55.44%)	0.579	11
DT	None	None	10-fold	-	FA	48.44% (48.00% / 48.86%)	0.476	0
					MD	50.59% (53.37% / 47.81%)	0.508	0
					Combined	50.35% (52.69% / 48.01%)	0.504	0
		Grid search		10-fold	FA	50.90% (50.05% / 51.73%)	0.512	0
					MD	51.52% (51.11% / 51.91%)	0.531	0
					Combined	50.44% (49.05% / 51.82%)	0.518	0
	Statistical filter	None			FA	51.50% (54.92% / 48.06%)	0.510	0
					MD	52.36% (55.66% / 49.05%)	0.527	0
					Combined	50.35% (54.49% / 46.25%)	0.498	0
		Grid search			FA	51.55% (51.36% / 51.72%)	0.519	0
					MD	53.95% (59.54% / 48.38%)	0.546	1
					Combined	50.73% (51.28% / 50.17%)	0.511	0
	Sequential elimination	None			FA	49.97% (49.59% / 50.38%)	0.485	0
					MD	51.90% (55.35% / 48.46%)	0.515	0
					Combined	48.93% (46.41% / 51.52%)	0.474	-

Note: Top accuracies for SVM, PLR and DT classifiers, and score for the best overall approach are highlighted in light blue. Optimisation with DT classifier, sequential feature elimination and combined feature set was only performed once (no repetitions), hence score not shown (section S2.2).

Table S19

Case-control classification accuracies and ROC AUC measures (on leave-one-out cross-validation) with *brain morphometric* features in cMDD-STR sample with replaced control participants (30 cases and 30 controls)

Classifier type	*Feature selection*	*Hyperparam. optimisation*	*Outer CV*	*Inner CV*	*Feature domain*	*Classification accuracy (sensitivity / specificity)*	*ROC AUC*
PLR	Embedded	Grid search	LOOCV	10-fold	Thickness	56.67% (53.33% / 60.00%)	0.583
					Surface area	43.33% (40.00% / 46.67%)	0.446
					Volume	38.33% (43.33% / 33.33%)	0.362
					Subcortical	53.33% (56.67% / 50.00%)	0.602
					Combined	61.67% (63.33% / 60.00%)	0.648
SVM	None	None	LOOCV	-	Thickness	56.67% (53.33% / 60.00%)	0.503
					Surface area	41.67% (36.67% / 46.67%)	0.439
					Volume	36.67% (50.00% / 23.33%)	0.304
					Subcortical	53.33% (53.33% / 53.33%)	0.639
					Combined	43.33% (50.00% / 36.67%)	0.486
		Grid search		LOOCV	Thickness	61.67% (60.00% / 63.33%)	0.570
					Surface area	35.00% (23.33% / 46.67%)	0.285
					Volume	31.67% (33.33% / 30.00%)	0.301
					Subcortical	60.00% (63.33% / 56.67%)	0.602
					Combined	48.33% (46.67% / 50.00%)	0.353
	Statistical filter	None			Combined	55.00% (50.00% / 60.00%)	0.539
		Grid search		10-fold	Combined	55.00% (56.67% / 53.33%)	0.564
	Sequential elimination	None			Thickness	53.33% (63.33% / 43.33%)	0.584
					Surface area	48.33% (46.67% / 50.00%)	0.492
					Volume	45.00% (63.33% / 26.67%)	0.360
					Subcortical	55.00% (50.00% / 60.00%)	0.636
DT	None	None	LOOCV	-	Thickness	51.67% (53.33% / 50.00%)	0.507
					Surface area	50.00% (60.00% / 40.00%)	0.438
					Volume	41.67% (50.00% / 33.33%)	0.477
					Subcortical	50.00% (50.00% / 50.00%)	0.456
					Combined	40.00% (50.00% / 30.00%)	0.305
		Grid search		LOOCV	Thickness	40.00% (53.33% / 26.67%)	0.158
					Surface area	56.67% (60.00% / 53.33%)	0.399
					Volume	46.67% (40.00% / 53.33%)	0.513
					Subcortical	43.33% (36.67% / 50.00%)	0.464
					Combined	30.00% (20.00% / 40.00%)	0.241
	Statistical filter	None			Combined	58.33% (76.67% / 40.00%)	0.441
		Grid search		10-fold	Combined	53.33% (56.67% / 50.00%)	0.492
	Sequential elimination	None			Thickness	46.67% (56.67% / 36.67%)	0.381
					Surface area	61.67% (63.33% / 60.00%)	0.501
					Volume	38.33% (36.67% / 40.00%)	0.351
					Subcortical	53.33% (66.67% / 40.00%)	0.454

Note: Top accuracies for SVM, PLR and DT classifiers are highlighted in light blue.

Table S20

Case-control classification accuracies and ROC AUC measures (on leave-one-out cross-validation) with *brain morphometric* features in cMDD-STR sample with added control participants (30 cases and 60 controls) and SMOTE oversampling of minority class in the training data

Classifier type	*Feature selection*	*Hyperparam. optimisation*	*Outer CV*	*Inner CV*	*Feature domain*	*Classification accuracy (sensitivity / specificity)*	*ROC AUC*
PLR	Embedded	Grid search	LOOCV	10-fold	Thickness	58.89% (46.67% / 65.00%)	0.540
					Surface area	45.56% (20.00% / 58.33%)	0.450
					Volume	50.00% (40.00% / 55.00%)	0.517
					Subcortical	55.56% (50.00% / 58.33%)	0.579
					Combined	64.44% (40.00% / 76.67%)	0.531
SVM	None	None	LOOCV	-	Thickness	67.78% (26.67% / 88.33%)	0.563
					Surface area	54.44% (13.33% / 75.00%)	0.398
					Volume	65.56% (23.33% / 86.67%)	0.478
					Subcortical	55.56% (46.67% / 60.00%)	0.567
					Combined	63.33% (10.00% / 90.00%)	0.590
		Grid search		LOOCV	Thickness	70.00% (10.00% / 100.00%)	0.500
					Surface area	66.67% (0.00% / 100.00%)	0.500
					Volume	65.56% (0.00% / 98.33%)	0.500
					Subcortical	67.78% (6.67% / 98.33%)	0.500
					Combined	66.67% (0.00% / 100.00%)	0.500
	Statistical filter	None			Combined	67.78% (53.33% / 75.00%)	0.702
		Grid search		10-fold	Combined	64.44% (13.33% / 90.00%)	0.556
	Sequential elimination	None			Thickness	65.56% (36.67% / 80.00%)	0.592
					Surface area	51.11% (10.00% / 71.67%)	0.393
					Volume	58.89% (23.33% / 76.67%)	0.461
					Subcortical	58.89% (46.67% / 65.00%)	0.568
DT	None	None	LOOCV	-	Thickness	52.22% (30.00% / 63.33%)	0.482
					Surface area	55.56% (30.00% / 68.33%)	0.478
					Volume	55.56% (30.00% / 68.33%)	0.421
					Subcortical	54.44% (36.67% / 63.33%)	0.365
					Combined	56.67% (36.67% / 66.67%)	0.396
		Grid search		LOOCV	Thickness	57.78% (43.33% / 65.00%)	0.434
					Surface area	63.33% (43.33% / 73.33%)	0.550
					Volume	52.22% (40.00% / 58.33%)	0.376
					Subcortical	51.11% (40.00% / 56.67%)	0.432
					Combined	51.11% (46.67% / 53.33%)	0.420
	Statistical filter	None			Combined	66.67% (53.33% / 73.33%)	0.583
		Grid search		10-fold	Combined	66.67% (60.00% / 70.00%)	0.568
	Sequential elimination	None			Thickness	55.56% (36.67% / 65.00%)	0.408
					Surface area	52.22% (33.33% / 61.67%)	0.389
					Volume	58.89% (33.33% / 71.67%)	0.458
					Subcortical	53.33% (36.67% / 61.67%)	0.388

Note: Top accuracies for SVM, PLR and DT classifiers are highlighted in light blue.
